# Remote near-field spectroscopy of vibrational strong coupling between organic molecules and phononic nanoresonators

**DOI:** 10.1038/s41467-022-34393-4

**Published:** 2022-11-11

**Authors:** Irene Dolado, Carlos Maciel-Escudero, Elizaveta Nikulina, Evgenii Modin, Francesco Calavalle, Shu Chen, Andrei Bylinkin, Francisco Javier Alfaro-Mozaz, Jiahan Li, James H. Edgar, Fèlix Casanova, Saül Vélez, Luis E. Hueso, Ruben Esteban, Javier Aizpurua, Rainer Hillenbrand

**Affiliations:** 1 CIC nanoGUNE BRTA, 20018 Donostia-San Sebastián, Spain; 2 Materials Physics Center, CSIC-UPV/EHU, 20018 Donostia-San Sebastián, Spain; 3 Donostia International Physics Center (DIPC), Donostia-San Sebastián, Spain; 4 Tim Taylor Department of Chemical Engineering, Kansas State University, Manhattan, KS 66506 USA; 5 IKERBASQUE, Basque Foundation for Science, 48009 Bilbao, Spain; 6 IFIMAC-Condensed Matter Physics Center, Instituto Nicolás Cabrera, and Departamento de Física de la Materia Condensada, Universidad Autónoma de Madrid, Madrid, E-28049 Spain; 7 CIC nanoGUNE BRTA and Department of Electricity and Electronics, UPV/EHU, 20018 Donostia-San Sebastián, Spain

**Keywords:** Near-infrared spectroscopy, Nanophotonics and plasmonics

## Abstract

Phonon polariton (PhP) nanoresonators can dramatically enhance the coupling of molecular vibrations and infrared light, enabling ultrasensitive spectroscopies and strong coupling with minute amounts of matter. So far, this coupling and the resulting localized hybrid polariton modes have been studied only by far-field spectroscopy, preventing access to modal near-field patterns and dark modes, which could further our fundamental understanding of nanoscale vibrational strong coupling (VSC). Here we use infrared near-field spectroscopy to study the coupling between the localized modes of PhP nanoresonators made of h-BN and molecular vibrations. For a most direct probing of the resonator-molecule coupling, we avoid the direct near-field interaction between tip and molecules by probing the molecule-free part of partially molecule-covered nanoresonators, which we refer to as remote near-field probing. We obtain spatially and spectrally resolved maps of the hybrid polariton modes, as well as the corresponding coupling strengths, demonstrating VSC on a single PhP nanoresonator level. Our work paves the way for near-field spectroscopy of VSC phenomena not accessible by conventional techniques.

## Abstract (web summary)

Vibrational strong coupling (VSC) promises ultrasensitive IR spectroscopy and modification of material properties. Here, nanoscale mapping of VSC between organic molecules and individual IR nanoresonators is achieved by remote near-field spectroscopy.

## Introduction

Strong coupling between molecular vibrations and infrared photons (vibrational strong coupling, VSC) leads to hybrid light-matter states^1–6^. They offer intriguing possibilities for ultra-sensitive vibrational spectroscopy^7–10^ and for modifying chemical reactions^3,4^. Typically, VSC is achieved with molecules embedded into microcavities, implying large mode volumes and large amounts of molecules, which limits access to quantum phenomena that may be accesible only for nanoscale amounts of molecules or at the level of a few molecules. In this regard, plasmonic infrared resonators are a promising route to achieve VSC at the nanoscale^7,8^ owing to their dramatically reduced mode volumes as compared to microcavities. Alternatively, phonon polaritons (PhP) can be employed for VSC experiments, offering stronger polariton confinement and larger quality factors^9–11^. Unfortunately, the far-field extinction cross-section of individual PhP nanoresonators^12–20^ is extremely small (due to their small size compared to the infrared wavelength), challenging infrared far-field spectroscopy. Further, subradiant dark modes—offering the advantage of longer lifetimes—are difficult to probe by far-field spectroscopy. These problems can be circumvented by nanoscale Fourier Transform Infrared (nano-FTIR) spectroscopy, which employs the strong field concentration at the apex of a metallic scanning probe tip (the near-field probe)^21,22^ to enable near-field spectroscopy and spatial mapping of both bright and dark modes of individual phononic nanoresonators^17,18^. However, the near-field mapping of VSC employing PhP nanoresonators has been elusive so far.

Nano-FTIR spectroscopy has been employed to study the coupling between molecular vibrations and plasmonic resonators, but the near-field probe itself can also couple with the plasmonic resonator and the molecules^7,23,24^, eventually reaching strong tip-resonator and tip-molecule coupling. Although this coupling may be exploited for on-demand control of VSC, it may challenge the probing of the hybrid polariton modes that are exclusively formed by the molecule-nanoresonator coupling.

In ref. 17, nano-FTIR spectroscopy was applied to study strong coupling between molecular vibrations and propagating PhPs on extended, unstructured h-BN layers. In this experiment, polariton interferometry had to be applied to obtain the polariton momenta and thus the polariton dispersion. This measurement principle and its associated data analysis offers the advantage that the tip-molecule and tip-polariton coupling does not affect the polariton dispersion and analysis of VSC. However, such experiments do not allow for analyzing the coupling between molecular vibrations and localized PhP modes, which will be of utmost importance to experimentally explore, test and verify future VSC concepts employing individual PhP nanoresonators.

Here we demonstrate that hybrid polariton modes formed by vibrational strong coupling between a single PhP nanoresonator and molecular vibrations can be studied and imaged in real space by nano-FTIR spectroscopy. We minimize the influence of the tip by probing the molecule-free part of partially molecule-covered PhP nanoresonators with a non-resonant metallic tip, which we refer to as remote near-field probing. We also verify our experimental results via comparative numerical simulations, where the near-field probe is modeled either by a point-dipole source (representing a non-disturbing near-field probe) or by an oscillating metal tip (representing a more realistic and potentially disturbing near-field probe).

## Results

### Nano-FTIR spectroscopy of half-covered phononic nanoresonators

**Fig. 1:**
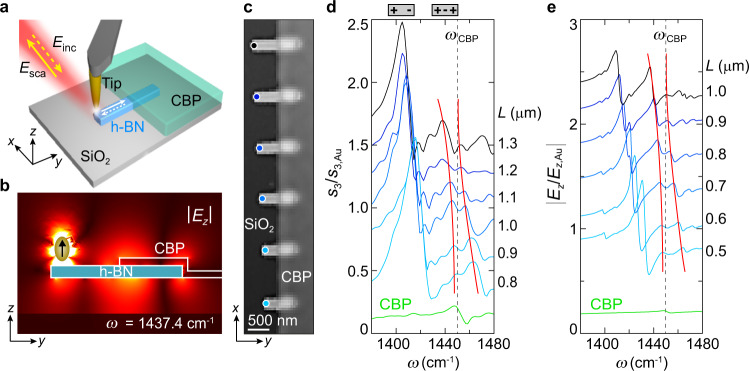
Tip-enhanced near-field probing of half molecule-covered h-BN nanoresonators. **a** Illustration of the experiment. **b** Numerical simulation of the electric field distribution at *ω* = 1437.4 cm^−1^ around an h-BN nanorod of 1000 nm length, 250 nm width and 87 nm height, whose right half is covered by a 50 nm thick CBP layer. The vertical arrow indicates a point-dipole source mimicking the tip. **c** Topography image of h-BN nanorods (length *L*, 87 nm height and 250 nm width) that are half covered by a 50 nm thick CBP layer. **d** Experimental nano-FTIR amplitude spectra recorded at the positions marked in panel **c** by dots of the respective color. For better visualization, spectra for *L* = 1.0 and 1.3 μm are scaled by a factor of 0.7. **e** Simulated nano-FTIR amplitude spectra, as explained in Fig. 3. For the width and thickness of the h-BN rods we used the nominal experimental values. The lengths *L* were chosen such that the second-order FP mode tunes across the molecular vibrational resonance of the CBP molecules at *ω* = 1450 cm^−1^. We attribute the differences between *L* in the experiment and the simulation to fabrication uncertainties such as trapezoid-like rod cross sections.^9,10^ **d**, **e** Green curves show experimental and simulated nano-FTIR amplitude spectra of a 50 nm thick bare CBP layer on a 250 nm thick SiO_2_ on Si substrate. Red lines are guides to the eye and mark peak positions. Gray-dashed lines indicate the CBP vibrational resonance at *ω*_CBP_ = 1450 cm^−1^ whose line width is 6.4 cm^−1^ (ref. 11). All spectra are normalized to that obtained on a Au reference surface, and are offset. We note that in the experimental spectra (Fig. 1d) we observe several smaller peaks, e.g., in the frequency range between 1420 and 1440 cm^−1^. They can be attributed to higher-order PhP modes^10^. In the simulated spectra, where the near-field probe is described by a point-dipole source (Fig. 1e), these peaks are only partially seen. A much better reproduction of these peaks is obtained in numerical simulations, where the near-field probe is described by a conical metallic tip (for further details and discussion see Supplementary Note 4).

Our experiment is illustrated in Fig. 1a. Half of a hexagonal boron nitride (h-BN) nanorod is covered with CBP molecules (4,4′-bis(N-carbazolyl)−1,1′-biphenyl; organic semiconductor) that exhibit a vibrational resonance at *ω*_CBP_ = 1450 cm^−1^ (Supplementary Note 1), as can be recognized from the near-field spectrum of a thin CBP layer shown by the green curve in Fig. 1d, e. The near-field probe, the non-resonant metallic tip^25^ of an atomic force microscope, is placed remotely with respect to the molecules at the opposite extremity of the h-BN rod. The tip (oscillating normal to the sample at frequency Ω) concentrates an illuminating broadband infrared laser beam at its apex to a nanoscale near-field spot, which excites phonon polaritons^26–28^ exhibiting Fabry-Perot (FP) resonances^9,18,19^ in the h-BN nanorod^17^. Figure 1b shows a simulation of the second-order FP mode excited by the near fields of a point dipole (note that this mode is often referred to as a dark mode, since it cannot be excited by far-field illumination due to its zero net electric dipole moment; see Supplementary Note 2). The coupling between the resonator mode and the layer of molecules—resulting from the strong overlap between the near field of the resonator mode and the molecules—is probed by recording of the tip-scattered field with an asymmetric Fourier transform spectrometer, yielding both near-field amplitude and phase spectra, *s*_3_(*ω*) and *φ*_3_(*ω*), respectively, where the index indicates that the detector signal was demodulated at 3Ω to suppress background signals (nano-FTIR spectroscopy; see Methods).

**Fig. 2:**
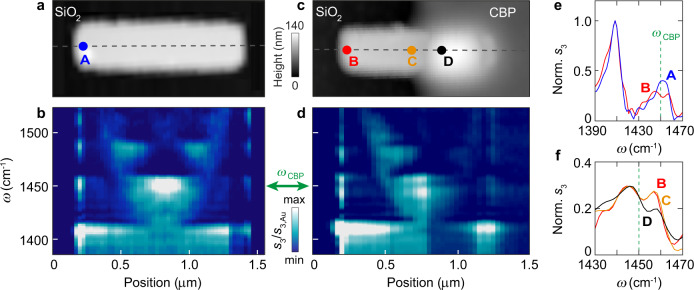
Nano-FTIR line scans of bare and half molecule-covered h-BN nanoresonators. **a** Topography image of a bare h-BN nanorod of 1.25 μm length, 110 nm height and 250 nm width. **b** Nano-FTIR amplitude spectra recorded along the horizontal dashed black line in panel **a**. **c** Topography image of an h-BN nanorod of 1.1 μm length, 87 nm height and 250 nm width, which is half covered with a 50 nm thick CBP layer. **d** Nano-FTIR amplitude spectra recorded along the horizontal dashed black line in panel **c**. **e** Nano-FTIR amplitude spectra of the bare (blue) and molecule-covered (red) h-BN nanorod at positions marked by blue and red dots in panels **a** and **c**, respectively. Both spectra are normalized to the peak maximum at 1408 cm^−1^. **f** Nano-FTIR amplitude spectra of the molecule-covered h-BN nanorod at positions marked by red, orange and black dots in panel **c**. The spectra are normalized to the peak maximum at 1445 cm^−1^. The vertical green dashed line marks the frequency of the molecular vibrational resonance of CBP.

**Fig. 3:**
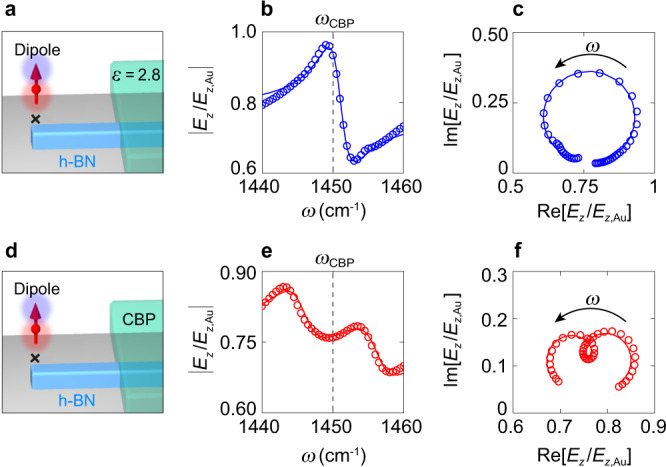
Theoretical description of near-field probing of VSC. **a** Simulation geometry, showing a point-dipole source above an h-BN nanorod of 785 nm length, 87 nm height and 250 nm width, which is half covered with a 50 nm thick layer of a permittivity *ε* = 2.8. **b** Amplitude of the *z*-component of the electric field below the dipole at the position marked by a cross in panel **a** (normalized to that obtained on a Au reference surface, $\left|{E}_{{{{{{\rm{z}}}}}}}/{E}_{{{{{{\rm{z}}}}}},{{{{{\rm{Au}}}}}}}\right|$), as function of frequency *ω*. **c** *z*-component of the electric field below the dipole source at the position marked by a cross in panel **a**, plotted in the complex plane. The arrow indicates increasing frequency *ω*. **d** Simulation geometry, showing a point-dipole source above the same h-BN nanorod as in panel **a** but half covered with CBP molecules. **e** Same as panel **b**, but the h-BN nanorod is half covered by a 50 nm thick CBP layer. **f** Same as panel **c**, but for a half CBP-covered h-BN nanorod. **b**, **c**, **e**, **f** Open symbols show simulation results. Solid lines show fits obtained with **b**, **c** a single harmonic oscillator model and **e**, **f** a coupled harmonic oscillator model describing the coupling between the nanoresonator modes and the molecular vibrations.

A topography image of the set of h-BN nanorods of 250 nm width, 87 nm height and various lengths *L* is shown in Fig. 1c, where one can also see the thin, homogeneous CBP layer of 50 nm thickness covering the right half of all resonators. The experimental nano-FTIR amplitude spectra (Fig. 1d; phase spectra are shown in Supplementary Fig. 3) recorded on the left rod extremity (measurement position marked by dots in Fig. 1c) clearly show the resonance peaks of the first and second-order FP resonances (illustrated by the schematic above the diagram in Fig. 1d and verified by the experimental mode pattern shown in Fig. 2), which shift to higher frequencies when the nanoresonator length *L* is reduced from 1300 to 800 nm. However, the peak of the second order mode does not cross the CBP vibrational resonance at *ω*_CBP_ = 1450 cm^−1^. Tracing the peak positions (marked by red lines) indeed reveals anti-crossing behavior, indicating that the nanoresonator near field couples with the molecular vibrations of the CBP layer. Most important, the nanoresonator-molecule coupling (occurring on the right half of the h-BN nanorods) can be well probed when the tip is placed on the left nanorod extremity, that is several 100 nm away from the molecules, where a direct near-field interaction between tip and molecules can be neglected (note that significant near-field interaction between tip and sample occurs only for distances smaller than the tip apex radius, here about 25 nm). The experimental near-field spectra can be well reproduced by numerical simulations (Fig. 1e), where the tip is modeled as a point-dipole source, which are described in more detail in Fig. 3.

### Spatio-spectral near-field mapping of phononic nanoresonators

Remote near-field spectroscopy can be applied for nanoscale spatial mapping of the resonator-molecule coupling via hyperspectral nanoimaging—even for dark modes that are not accessible by far-field spectroscopy. In the future, such a possibility could be applied, for example, to study advanced resonator structures where a variety of different resonator modes may coexist and couple with the molecular vibrations. In Fig. 2 we apply this capability for direct experimental identification of the phononic resonator mode that couples with the molecular layer and to verify that the peak splitting is caused by the presence of the molecules. To that end, we recorded nano-FTIR amplitude spectra along the principal axis of a bare (Fig. 2a, b) and a half-covered (Fig. 2c, d) h-BN nanoresonator. The lengths *L* of the nanoresonators were chosen such that their second-order FP resonance occurs at the molecular vibrational resonance frequency, *ω*_CBP_ = 1450 cm^−1^. For each spectral peak we observe strong near-field oscillations along the principal nanoresonator axis. The number of oscillations increases steadily with increasing frequency, revealing a series of longitudinal FP modes. The three near-field maxima (in the center and at the two extremities of the nanoresonator) at 1450 cm^−1^ clearly reveal the second-order (dark) FP mode. More important, the second-order FP modal near-field pattern of the half-covered nanoresonator exhibits a small spectral dip at the molecular vibrational resonance of CBP at 1450 cm^−1^ (dark stripe in Fig. 2d) all along the principal resonator axis, which is absent in the hyperspectral linescan of the bare nanoresonator (Fig. 2b). For a better quantitative comparison, we show in Fig. 2e the nano-FTIR amplitude spectra recorded at the left extremity of the bare and molecule-covered h-BN nanoresonators (positions marked by blue and red dots in Fig. 2a, c, respectively). We clearly see that the peak of the first-order FP mode (far away from the CBP resonance at* ω*_CBP_ = 1450 cm^−1^) is nearly the same for both nanoresonators, whereas the peak of the second-order FP mode of the molecule-covered h-BN nanoresonator exhibits a spectral dip occurring at *ω*_CBP_, as typical for the coupling between molecular vibrations and nanoresonator modes^7,9,10,23^. The spatio-spectral observation presented in Fig. 2 demonstrates that the nanoresonator-molecule coupling can in principle be probed at any location where the nanoresonator mode can be activated by the near-field probe. On the other hand, Fig. 2d reveals that the near-field signal on the molecule-covered part of the nanoresonator is strongly reduced due to the increased tip-nanoresonator distance and that the spectral line shape is modified due to the direct near-field interaction between tip and molecules, highlighting the advantages of probing the molecule-free part of the resonator, that is, probing larger near-field signals and reduction of undesirable near-field interaction between tip and molecules. The modification of the line shape is more clearly seen in Fig. 2f, which compares spectra recorded on the molecule-free nanoresonator part (B and C) with spectrum D that was recorded on the molecule-covered part. Specifically, we see that the spectrum D exhibits an asymmetric line shape and that the dip is shifted to slightly higher frequencies. This asymmetric line shape can be explained as superposition of the symmetric nano-FTIR spectrum of the molecule-covered nanoresonator (B) and the asymmetric nano-FTIR spectrum of a bare CBP layer (such as the green spectrum in Fig. 1d) caused by the direct near-field interaction between tip and molecules. The later exhibits a derivative-like spectral line shape, that is, a peak and a dip to the left and right of the molecular resonance, respectively, which is a typical characteristic of nano-FTIR amplitude spectra of molecular layers^29^.

### Theoretical description of remote near-field probing of VSC

To understand how the hybrid nanoresonator-molecule modes manifest in the near-field spectra, we first discuss in Fig. 3 numerical simulations where the tip is modeled as a point-dipole source^17,30^ (red arrow in Fig. 3a, d) located above the h-BN resonator. We obtain complex-valued near-field spectrum by evaluating the vertical (*z-*) component of the electric field below the dipole (evaluation position marked by a cross in Fig. 3a, d) as a function of frequency, *E*_z_(*ω*). Further simulation details are described in the Methods section. Importantly, the dipole moment of the source is kept constant and consequently is not modified by the fields of the nanoresonator. This simplified modeling of the tip allows for excluding a potential coupling of the tip with the nanoresonator and the molecules, and thus exclusively reveals the spectral near-field signature of the coupling between the nanoresonator and the molecular vibrations.

Figure 3b shows the simulated near-field amplitude spectrum |*E*_z_(*ω*)| (open symbols), of a h-BN nanorod that is half covered with a layer of permittivity *ε* = 2.8 (corresponding to the permittivity of CBP without the molecular resonance at 1450 cm^−1^). Its length was chosen such that the second-order FP resonance is at *ω* = 1450 cm^−1^, which manifests as a single peak in the near-field amplitude spectrum. Repeating the simulation when the h-BN nanorod is half covered by a CBP layer (Fig. 3e), the nanoresonators’ near-field amplitude peak splits into two peaks (open symbols), which is a direct consequence of the coupling between the nanoresonator mode and molecular vibrations. For quantifying the coupling between the h-BN nanoresonator and the molecular vibrations, we fit the simulated near-field spectra by a model of two coupled harmonic oscillators (Methods), which represent the nanoresonator mode and the molecular vibration with eigenfrequencies *ω*_*i*_ and damping parameters *γ*_*i*_ (the index *i* denotes either PhP or CBP). The two oscillators are coupled to each other through the coupling strength *g*. For a most reliable analysis, we performed complex-valued fitting of the simulated near-field spectra ${E}_{{{{{{\rm{z}}}}}}}\left(\omega \right)=\left|{E}_{{{{{{\rm{z}}}}}}}\left(\omega \right)\right|{e}^{i\varphi \left(\omega \right)}$, where |*E*_z_(*ω*)| and *φ*(*ω*) are the amplitude and phase spectra (Fig. 3f). Plotting the fits by red solid lines in Fig. 3e, f, we find an excellent agreement with the simulated spectra (red open symbols), which confirms the validity of the two coupled harmonic oscillator model to fit the simulated near-field spectra.

Before analysing the experimental nano-FTIR spectra, we note that the simulated near-field spectrum of the h-BN nanoresonator in absence of molecular vibrations yields a nearly circular trajectory in the complex plane (Fig. 3c). Interestingly, when the nanoresonator is half covered with CBP molecules, the topology of the trajectory of the complex-valued near-field spectra changes. We find that a small loop appears within the main circular trajectory (Fig. 3f), which reveals the coupling between the resonator mode and the molecular vibrations, i.e., that not only a mere superposition of two modes is observed, (Supplementary Note 5), similar to a recent observation in far-field ellipsometry of excitonic coupling in classical microresonators^31^. The topology change of complex-valued trajectories may become an interesting means for characterizing coupling phenomena.

### Quantitative analysis of experimental nano-FTIR spectra

**Fig. 4:**
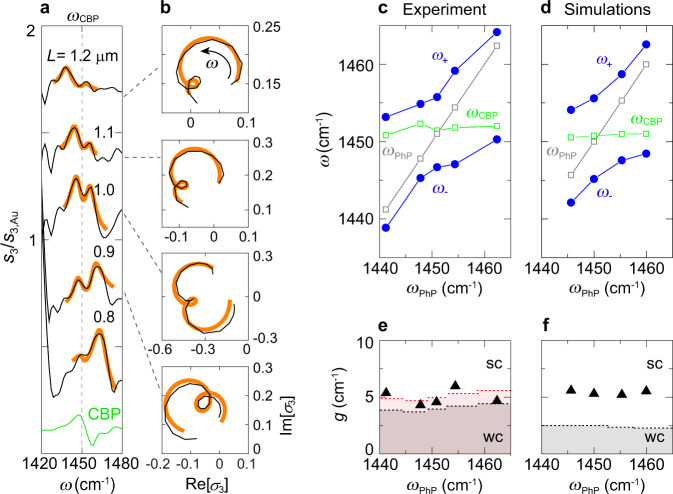
Quantitative analysis of nano-FTIR spectra by coupled harmonic oscillator fitting. **a** Black curves show the experimental nano-FTIR amplitude spectra of h-BN nanorods of length *L*, which are half covered with CBP molecules (same data as in Fig. 1d). Orange curves show fits obtained using the coupled harmonic oscillator model. For better visualization, the spectrum for *L* = 1.0 μm is scaled by a factor of 0.7. **b** Black curves show the complex-valued experimental nano-FTIR spectra, ${\sigma }_{3}\left(\omega \right)={s}_{3}\left(\omega \right){e}^{i{\varphi }_{3}\left(\omega \right)}$, plotted in the complex plane. Orange curves show fits obtained using the coupled harmonic oscillator model. **c** Eigenfrequencies *ω*_±_ of the hybrid modes, the nanoresonators´ bare eigenfrequencies *ω*_PhP_ (gray squares), and bare molecular vibrational eigenfrequency *ω*_CBP_ (green squares), all of them obtained by fitting of the complex-valued experimental nano-FTIR spectra. **d** Same as panel **c**, but obtained by fitting complex-valued simulated near-field spectra of h-BN nanoresonators of length *L* = 1.2 to 0.8 μm, which are half covered by CBP molecules (the corresponding amplitude spectra are shown in Fig. 1e; the complex-valued spectra are shown in Supplementary Fig. 3). The tip is modeled by a point-dipole source as in Fig. 3. **e**, **f** Coupling strength *g* obtained from the fitting of the experimental and simulated near-field spectra, respectively. Black and red dashed horizontal lines indicate the transition from weak (WC) to strong (SC) coupling defined by *g* = |*γ*_PhP_ + *γ*_CBP_ | */*4 ref. 32 and *g* = |*γ*_PhP_ + *γ*_CBP_ | /3.23 (see discussion below), respectively.

For analysing the experimental near-field spectra (Fig. 4a, b), we first assume that the tip is solely illuminating the nanoresonator (i.e., that it acts like a dipole source with a constant dipole moment and for that reason does not need to be modeled as another coupled oscillator). We thus apply the same model of two coupled harmonic oscillators as for the simulated near-field spectra to fit the experimental spectra. We further assume that the PhP mode is not excited by far-field illumination because it is a dark mode. Figure 4b shows the experimental near-field spectra of the half-covered nanoresonators in the complex plane (black lines), as well as the excellent fitting results (orange lines). The corresponding amplitude and phase spectra are shown in Fig. 4a and Supplementary Fig. 3, respectively. The small loops in the complex plane clearly reveal the coupling between the nanoresonator mode and the molecular vibrations, as predicted by the simulations in Fig. 3f. We note that the size of the small loop slightly varies in the experimental spectra of the different nanoresonators, which we attribute to variations of the resonators´ quality factor due to fabrication uncertainties. From the fits we determined for each experimental near-field spectrum (i.e. for each nanoresonator) the coupling strength *g*, the PhP eigenfrequency *ω*_PhP_ and damping *γ*_PhP_, the molecular vibrational eigenfrequency *ω*_CBP_ and damping *γ*_CBP_ (see Methods and Supplementary Table 2). From these parameters we calculated the eigenfrequencies of the new hybrid modes (see Methods) for each nanoresonator, *ω*_±_, which are shown in Fig. 4c (blue symbols) as a function of the nanoresonators´ eigenfrequencies *ω*_PhP_. We observe a clear anti-crossing of the hybrid modes, which indicates strong coupling. To determine the coupling regime for each individual resonator, we mark in Fig. 4e the transition from weak to strong coupling—defined by the standard criterium *g* = |*γ*_PhP_ + *γ*_CBP_ | /4 (ref. 32)—by black dashed horizontal lines. Interestingly, we find that *g* is above the black dashed line for all individual resonators, indicating that all of them are strongly coupled with the molecular vibrations.

Importantly, applying the same fitting procedure to the simulated complex-valued near-field spectra (where the tip is modeled as a point-dipole source; see Methods and Supplementary Note 3) yields hybrid eigenmodes (Fig. 4d) and coupling strengths (Fig. 4f) that match well the experimental results. Since the tip is not involved in the nanoresonator-molecule coupling in the simulations, this quantitative agreement (for further discussion see Supplementary Note 6) lets us further assume that the coupling between the PhP mode and the molecular vibrations in our experiment can be well described with a simple two coupled harmonic oscillator model, without the need of considering the tip as a third oscillator. Our analysis thus demonstrates the capability of remote near-field spectroscopy to probe the hybrid modes arising from the coupling between a single PhP nanoresonator and nanoscale amounts of organic molecules.

We note that the damping *γ*_PhP_ obtained from the simulated near-field spectra is a factor of three lower than that obtained from the experimental spectra (see Supplementary Note 3). We explain this finding by PhP scattering and absorption at fabrication-induced irregularities and damage of the h-BN nanorod edges^9,10^, which is not considered in the simulation. Although the damping has only a minor influence on the coupling strength *g* and on the determination of the hybrid eigenmodes (see Eq. (10) in Methods), it is a crucial parameter that determines whether a coupled system is in the weak or in the strong coupling regime. Marking the transition from weak to strong coupling in Fig. 4f by black horizontal dashed lines (analogue to Fig. 4e), we find that it occurs at much lower *g* for the simulation as compared to experiment, which shows that in simulations we are deeper inside the strong coupling regime than in the experiment.

### Influence of the oscillating tip and signal demodulation

So far, we have not explicitly considered in the simulations that the tip is a long metallic cone, that the tip is oscillating, and that the detector signal is demodulated at higher harmonics of the tip oscillation frequency. To elucidate the influence and impact of these key features on the spectral mode positions and linewidths, we performed numerical simulations where the tip is modeled as a metal cone and the tip oscillation and signal demodulation are considered. The results are shown in the Supplementary Note 6 and 7 and summarized as follows. We first applied the coupled harmonic oscillator model (same as used in Fig. 3) to fit the simulated near-field spectra where the tip is modeled as a metal cone but without considering the tip oscillation and signal demodulation. Compared to the fitting parameters obtained from the unperturbed molecule-covered resonator spectra (obtained from the point-dipole source simulations), we find that the presence of the metallic tip yields negligible spectral shifts of the bare h-BN nanoresonator mode and the hybrid polariton modes (<3 cm^−1^), a negligible change of the sum of damping parameters, *γ*_PhP_ + *γ*_CBP_, and only a slight reduction of the coupling strength by a factor of about 1.26. In a second simulation, we additionally implemented tip oscillation and signal demodulation. Fitting of the simulated spectra yields nearly the same coupling strengths and mode positions as before. However, for the sum of the damping parameters, *γ*_PhP_ + *γ*_CBP_, we obtain values that are reduced by a factor of about 1.56 as compared to the results obtained from the simulations where the tip is modelled as a point-dipole source. Since the presence of the tip, its oscillation and signal demodulation can yield significantly different values for *g* and *γ*_PhP_ + *γ*_CBP_, we re-evaluate the coupling regime obtained in the experiment according to a modified condition, 1.26 *g*/[1.56(*γ*_PhP_ + *γ*_CBP_)] > 0.25, where *g* and *γ*_PhP_ + *γ*_CBP_ are the parameters (listed in Table 1) obtained by fitting the experimental near-field spectra. We find that two of the nanoresonators (length *L* = 1.2 µm and *L* = 0.9 µm) satisfy the strong coupling criteria, whereas the other nanoresonators are at the onset of strong coupling. The transition from weak to strong coupling- according to this re-evaluation—is marked in Fig. 4e by red dashed horizontal lines. Since the modified condition for evaluating the coupling regime is more conservative than the original one (*g*/[*γ*_PhP_ + *γ*_CBP_] > 0.25), the transition occurs in Fig. 4e at larger *g* values. We note that the factors used to re-evaluate the coupling regime are specific for our study and may change for different tip oscillation amplitudes or signal demodulation orders.

**Table 1:** Parameters obtained by fitting the experimental complex-valued spectra shown in Fig. 4b via the coupled harmonic oscillator model

*L* (µm)	*ω*_PhP_	*γ*_PhP_	*ω*_CBP_	*γ*_CBP_	*g*	*γ*_PhP_ + *γ*_CBP_	$\frac{1.26g}{1.56({\gamma }_{{{{{{\rm{PhP}}}}}}}+{\gamma }_{{{{{{\rm{CBP}}}}}}})}$
1.2	1441.2	9.4	1450.8	6.0	5.4	15.4	0.28
1.1	1447.8	8.9	1452.3	6.0	4.3	14.9	0.23
1.0	1451.0	8.8	1451.4	7.0	4.6	15.8	0.23
0.9	1454.4	10.2	1451.8	6.6	6.0	16.8	0.29
0.8	1462.4	10.7	1452.0	7.0	4.7	17.7	0.21

All parameters from the second to the seventh column are expressed in cm^−1^. Errors according to the standard deviation obtained by fitting the individual spectra are presented in Supplementary Table 2.

## Discussion

In summary, we demonstrated that infrared near-field spectroscopy can be applied for nanoscale spatial mapping of strong coupling between molecular vibrations and resonating dark PhP modes. To that end, we introduced the concept of remote near-field probing with a non-resonant tip, where the tip and molecules are separated such that the direct near-field interaction between tip and molecules is avoided. Such minimal-invasive probing offers the advantage that the hybrid nanoresonator-molecule modes and coupling strengths can be determined within the model of two coupled harmonic oscillators without considering the tip as a third oscillator, which significantly simplifies the coupling analysis and increases its robustness. On the other hand, numerical simulations of remote near-field spectroscopy including the metal tip, its oscillation and signal demodulation reveal that damping parameters may be underestimated when tip oscillation and signal demodulation are not taken into account for fitting the near-field spectra with a coupled harmonic oscillator model.

Since near-field spectroscopy is available in the wide spectral range between visible and terahertz frequencies, we envision remote near-field probing studies of strong coupling of different plasmonic and phononic resonators (which apart from h-BN could be made, for example, from VO_5_ or MoO_3_ that cover various infrared frequency ranges) with molecular vibrations or excitons in various resonator geometries. The possibility to probe dark modes and spatially control the selective excitation and probing of coexisting modes could pave the way to explore strong coupling configurations that are not accessible by far-field spectroscopy.

## Methods

### Growth of monoisotopic ^10^B h-BN crystals

The growth of ^10^B enriched h-BN crystals was done by the metal flux method^33^. Powders of 40 g Fe, 25 g Cr, 1 g ^10^B were mixed in an alumina crucible and placed in a single-zone furnace. After a dwell time of 24 h at 1550 °C, the furnace was cooled at a rate of 4 °C/h to 1400 °C, to precipitate the h-BN crystals on the metal surface. This was subsequently followed by quick quenching to room temperature.

### Fabrication of h-BN nanoresonators

We first mechanically exfoliated ^10^B enriched h-BN crystals using blue Nitto tape (Nitto Denko Co., SPV 224 P). Then the flakes were exfoliated from the tape onto a transparent polydimethyl-siloxane (PDMS) stamp. Flakes of appropriate thickness and size were then transferred onto a Si/SiO_2_ (250 nm) substrate using the deterministic dry transfer technique^34^. For patterning the h-BN nanoresonators we used high-resolution electron beam lithography using a double-layer poly(methyl methacrylate) (PMMA) resist (495 A4/950 A2). After development of the resist, a 3 nm-thick Cr layer was deposited onto the sample by e-beam evaporation, followed by thermal evaporation of 40 nm of Al. After lift-off in acetone, chemical etching of the h-BN flake was performed using a SF6/Ar 1:1 plasma mixture at 20 sccm flow, 100 mTorr pressure and 100 W power for 60 s (RIE OXFORD PLASMALAB 80 PLUS reactive ion etcher). For removing the Cr-Al mask from the h-BN structures, the sample was immersed in a chromium etchant (Sigma-Aldrich Co., 651826) for 20 min. After rinsing in deionized water, the sample was dried using a N_2_ gun.

### Thermal evaporation of CBP

4,4′-bis(N-carbazolyl)-1,1′-biphenyl with sublimed quality (99.9%) (Sigma Aldrich) was thermally evaporated in an ultra-high vacuum evaporator chamber (base pressure <10^−9^ mbar), at a rate of 0.1 nm s^−1^ using a Knudsen cell.

### Preparation of a diamond mask for structured CBP deposition

A diamond mask was used for depositing the thermally evaporated CBP molecules only on one half of the h-BN nanoresonators. The mask was cut from a polycrystalline diamond film (500 nm thick) by focused Ga-ion beam (FIB) milling in a Helios 450S (FEI) dual beam system (milling conditions: 30 kV and 9.3 nA). Using an in situ platinum deposition, the diamond mask was attached to the tungsten tip of an Omniprobe micromanipulator and physically placed on top of the nanoresonators such that it covered half of each. The mask was fixed on the sample surface by in situ Pt-deposition. After CBP deposition by thermal evaporation (see above), the Omniprobe micromanipulator was approached to the mask. The mask was fixed to the Omniprobe by in situ Pt deposition. By FIB milling, the mask was detached from the sample and could be removed via the Omniprobe micromanipulator.

### Nano-FTIR spectroscopy

We used a commercial scattering-type scanning near-field optical microscope (s-SNOM) setup comprising a nano-FTIR module (Neaspec/Attocube, Germany), in which the oscillating (at a frequency Ω ≅ 270 kHz) Pt/Ir-coated AFM tip (Arrow-NCPt-50, Nanoworld, Nano-World AG, Neuchâtel, Switzerland) was illuminated by p-polarized mid-IR broadband radiation generated by a supercontinuum laser (average power of about 0.5 mW; frequency range 1200–1700 cm^−1^). The detector signal was demodulated at a frequency 3 Ω for effective background suppression. Interferograms were measured by recording the demodulated detector signal as a function of the position of the reference mirror, *d*, at a fixed tip position. For apodization of the interferograms a Planck-taper window function with *ε* = 0.2 was applied. After zero-filling (4× padding) we Fourier transformed the interferograms to obtain complex-valued near-field point spectra, *E*_s_(*ω*). Each spectrum of Fig. 1d is an average of three spectra recorded in steps of 40 nm along the *y*-axis. We normalized the obtained point spectra to a reference spectrum recorded on gold, *E*_s,Au_(*ω*). The spectral resolution was 6.25 cm^−1^.

### Numerical simulations where the tip is modeled as a point-dipole source

We performed the numerical simulations (Figs. 1e, 3, 4d, f) using the Radio Frequency Module of COMSOL Multiphysics software. This module solves Maxwell’s equations in the frequency domain based on the Finite Element Method (FEM). The h-BN nanoresonators were modeled as a rectangular structure of *w* = 250 nm width (along the *x*-direction), *d* = 87 nm thickness (along the *z*-direction) and variable length *L* (along the *y*-direction). The resonators are on top of a 250 nm thick layer with the permittivity of SiO_2_, which is on top of a Si substrate. The CBP layer was modeled as a 50 nm thick layer covering half the length of the h-BN nanorod. The material permittivities are provided below. To ensure numerical convergence of the simulated near-field spectra, the complete structure (point-dipole source, h-BN nanorod and CBP layer) was located in a homogeneous rectangular box (filled with air) of 8 × *w* width, 25 × *d* depth and 4 × *L* length. We use perfectly matched layers (PML) for the boundaries of the simulation box and free triangular elements for the nanorod mesh and free tetrahedral elements for all other structures.

To obtain the numerical results shown in Figs. 1e, 3, 4d, f, we modeled the tip as a point-dipole source oriented perpendicularly to the substrate (along the *z*-direction). This model assumes that the elongated tip in the experiment is oriented perpendicularly (*z*-direction) to the h-BN nanorod and is illuminated by p-polarized light. The experimental signal detected in the far field (*E*_*s*_) was approximated by the vertical component of the electric near field (*E*_*z*_) at an evaluation point below the point-dipole source. In all the calculations, the point-dipole and the evaluation points were located at coordinates (*x* = 0*, y* = *−L*/2 + 50 nm*, z* =  350 nm) and (*x* = 0*, y* = *−L/*2 + 50 nm*, z*  = 65 nm), respectively. We set the origin (*x* = 0*, y* = 0*, z* = 0) at the middle of the top surface of the nanorod. The CBP layer covered the nanorod for *y* > 0. For comparison with the experimental near-field spectra, the simulated near-field spectra were normalized to *E*_*z*_ obtained when the dipole source is located above a gold (i.e. reference) substrate.

### Permittivity of h-BN

The permittivity of the isotopically (^10^B) enriched h-BN (in-plane ${\varepsilon }_{{{{{{\rm{hBN}}}}}},\perp }$, out-of-plane ${\varepsilon }_{{{{{{\rm{hBN}}}}}},\parallel }$) is described by a Drude-Lorentz model^35^

1 $${\varepsilon }_{{{{{{\rm{hBN}}}}}},j}={\varepsilon }_{{{\infty }},j}\left(1+\frac{{\omega }_{{{{{{\rm{LO}}}}}},j}^{2}-{\omega }_{{{{{{\rm{TO}}}}}},j}^{2}}{{\omega }_{{{{{{\rm{TO}}}}}},j}^{2}-{\omega }^{2}-i\omega {\Gamma }_{j}}\right),$$

where *j* indicates the in-plane (⊥) or out-of-plane (||) component. In Eq. (1), *ω*_*TO,j*_ and *ω*_*LO,j*_ are the TO and LO phonon frequencies, ${\Gamma_j}$ is the damping constant and ${\varepsilon }_{\infty,j}$ is the high-frequency permittivity. The values used for each constant are presented in Table 2.

**Table 2:** Parameters for calculating the in-plane and out-of-plane permittivity components of h-BN according to Eq. (1)

	In-plane (*ε*_⊥_)	Out-of-plane (*ε*_||_)
*ε*_*∞,j*_	3	2.8
*ω*_TO,*j*_	1395 cm^−1^	785 cm^−1^
*ω*_LO,*j*_	1630 cm^−1^	845 cm^−1^
Γ_*j*_	2 cm^−1^	1 cm^−1^

### Permittivity of CBP

We model the permittivity of CBP as the sum of a non-resonant background plus four oscillators which describe the molecular vibrations of CBP within the frequency range of interest. Thus, the permittivity of CBP is given by^11^

2 $${\varepsilon }_{{{{{{\rm{CBP}}}}}}}={\varepsilon }_{{{\infty }},{{{{{\rm{CBP}}}}}}}+\mathop{\sum }\limits_{k=1}^{4}\frac{{\omega }_{p,k}^{2}}{{\omega }_{0,k}^{2}-{\omega }^{2}-i{\omega \Gamma }_{{{{{{\rm{CBP}}}}}},k}},$$

where ${\varepsilon }_{\infty,{{{{{\rm{CBP}}}}}}}=2.8$ is the high-frequency permittivity and *ω*_*p,k*_, *ω*_0*,k*_ and Γ_CBP,*k*_ are the strength, natural frequency, and damping constant of the *k*-th oscillator, respectively. The values used for each constant are presented in Table 3.

**Table 3:** Parameters for calculating the permittivity of CBP according to Eq. (2)

*k*	*ω*_0,*k*_ [cm^−1^]	*ω*_*p,k*_ [cm^−1^]	Γ_CBP,*k*_ [cm^−1^]
1	1450.0	128	6.4
2	1478.6	47	4.4
3	1500.1	91	9.4
4	1507.4	99	6.1

### Permittivity of Si, SiO_2_ and Au

For the numerical calculations shown in the main text, we considered the h-BN nanorod to be on top of a Si/SiO_2_ substrate. For the permittivity of Si, we used a constant value *ε*_si_ = 12. The SiO_2_ permittivity was approximated by the following analytical function

3 $${\varepsilon }_{{{{{{\rm{Si}}}}}}{{{{{{\rm{O}}}}}}}_{2}}=	 \, {a}_{0}+{a}_{1}{\lambda }_{0}+{a}_{2}{\lambda }_{0}^{2}+{a}_{3}{\lambda }_{0}^{3}+{a}_{4}{\lambda }_{0}^{4}+{a}_{5}{\lambda }_{0}^{5}+\frac{{a}_{6}}{{\left({\lambda }_{0}-{a}_{7}\right)}^{2}+{a}_{8}^{2}} \\ 	+ i\frac{{a}_{9}}{{\left({\lambda }_{0}-{a}_{7}\right)}^{2}+{a}_{10}^{2}},$$

with *λ*_0_ being the free space wavelength expressed in microns, *a*_0_ = 942.883, *a*_1_ = −712.749 μm^−1^, *a*_2_ = 215.563 μm^−2^, *a*_3_ = −32.5483 μm^−3^, *a*_4_ = 2.45449 μm^−4^, *a*_5_ = −0.0740283 μm^−5^, *a*_6_ = −0.1404 μm^2^, *a*_7_ = 8.078 μm, *a*_8_ = 0.1404 μm, *a*_9_ = −0.1426 μm^2^ and *a*_10_ = 0.1426 μm. The permittivity of Au was taken from ref. 36.

### Coupled harmonic oscillator model

To fit the data shown in Figs. 3 and 4, we described the coupling between the Fabry-Perot phonon polariton resonance and the molecular vibrations by a classical model of two coupled harmonic oscillators. The equations of motion that describe the coupled system are determined by the following relations^37–40^

4 $${\ddot{x}}_{{{{{{\rm{PhP}}}}}}}\left(t\right)+{\gamma }_{{{{{{\rm{PhP}}}}}}}{\dot{x}}_{{{{{{\rm{PhP}}}}}}}\left(t\right)+{\omega }_{{{{{{\rm{PhP}}}}}}}^{2}{x}_{{{{{{\rm{PhP}}}}}}}\left(t\right)-2g{\dot{x}}_{{{{{{\rm{CBP}}}}}}}\left(t\right)={F}_{{{{{{\rm{PhP}}}}}}}\left(t\right),$$

5 $${\ddot{x}}_{{{{{{\rm{CBP}}}}}}}\left(t\right)+{\gamma }_{{{{{{\rm{CBP}}}}}}}{\dot{x}}_{{{{{{\rm{CBP}}}}}}}\left(t\right)+{\omega }_{{{{{{\rm{CBP}}}}}}}^{2}{x}_{{{{{{\rm{CBP}}}}}}}\left(t\right)+2g{\dot{x}}_{{{{{{\rm{PhP}}}}}}}\left(t\right)={F}_{{{{{{\rm{CBP}}}}}}}\left(t\right),$$

where the dots denote time derivatives, *x*_PhP_(*t*) represents the PhP mode with resonance frequency *ω*_PhP_ and damping constant *γ*_PhP_, and *x*_CBP_(*t*) represents the molecular vibration of CBP with resonance frequency *ω*_CBP_ and damping constant *γ*_CBP_. *g* is the coupling strength between the two resonators and *F*_PhP_(*t*), *F*_CBP_(*t*) represent the effective forces that drive each resonator. The effective forces are proportional to the near fields provided by the tip. In particular, we set *F*_CBP_(*t*) = 0 as in our experiment the near fields of the tip do not act directly on the CBP molecules.

Through a time-to-frequency Fourier transform of Eqs. (4) and (5), we find the following steady-state solutions

6 $${\hat{x}}_{{{{{{\rm{PhP}}}}}}}\left(\omega \right)=\frac{{\omega }_{{{{{{\rm{CBP}}}}}}}^{2}-{\omega }^{2}-i{\gamma }_{{{{{{\rm{CBP}}}}}}}\omega }{\left({\omega }_{{{{{{\rm{PhP}}}}}}}^{2}-{\omega }^{2}-i{\gamma }_{{{{{{\rm{PhP}}}}}}}\omega \right)\left({\omega }_{{{{{{\rm{CBP}}}}}}}^{2}-{\omega }^{2}-i{\gamma }_{{{{{{\rm{CBP}}}}}}}\omega \right)-{\left(2g\omega \right)}^{2}}{\hat{F}}_{{{{{{\rm{PhP}}}}}}}\left(\omega \right),$$

7 $${\hat{x}}_{{{{{{\rm{CBP}}}}}}}(\omega )=\frac{i2g\omega }{\left({\omega }_{{{{{{\rm{PhP}}}}}}}^{2}-{\omega }^{2}-i{\gamma }_{{{{{{\rm{PhP}}}}}}}\omega \right)\left({\omega }_{{{{{{\rm{CBP}}}}}}}^{2}-{\omega }^{2}-i{\gamma }_{{{{{{\rm{CBP}}}}}}}\omega \right)-{\left(2g\omega \right)}^{2}}{\hat{F}}_{{{{{{\rm{PhP}}}}}}}\left(\omega \right),$$

with ${\hat{x}}_{{{{{{\rm{PhP}}}}}}}\left(\omega \right){=}{{{{{\mathscr{F}}}}}}[{x}_{{{{{{\rm{PhP}}}}}}}\left(t\right)]$, ${\hat{x}}_{{{{{{\rm{CBP}}}}}}}\left(\omega \right){=}{{{{{\mathscr{F}}}}}}[{x}_{{{{{{\rm{CBP}}}}}}}\left(t\right)]$, ${\hat{F}}_{{{{{{\rm{PhP}}}}}}}\left(\omega \right){=}{{{{{\mathscr{F}}}}}}[{F}_{{{{{{\rm{PhP}}}}}}}\left(t\right)]$ where ${{{{{\mathscr{F}}}}}}$ is the time-to-frequency Fourier transform.

### Fitting of harmonic oscillator model to near-field spectra

To fit the simulated spectra (Figs. 3 and 4d), we used Eq. (6) plus a complex-valued offset term (*x*_0_ + *y*_0_*i*), which accounts for the non-polaritonic near-field interaction between the tip and the nanoresonator:

8 $${\hat{x}}_{{{{{{\rm{sim}}}}}}}\left(\omega \right)={\hat{x}}_{{{{{{\rm{PhP}}}}}}}\left(\omega \right)+{x}_{0}+{y}_{0}i.$$

To fit the experimental spectra (Fig. 4a, b), we multiply Eq. (6) by a complex-valued factor (*e*^*iϕ*^) that accounts for the drift of the interferometer between the measurement of the nanoresonator spectra and the reference measurement on a flat gold surface:

9 $${\hat{x}}_{{{\exp }}}\left(\omega \right)={\hat{x}}_{{{{{{\rm{PhP}}}}}}}\left(\omega \right){e}^{i\phi }+{x}_{0}+{y}_{0}i.$$

In the fitting procedure, we used as free parameters *ω*_PhP_, *γ*_PhP_, *g*, ${\hat{F}}_{{{{{{\rm{PhP}}}}}}}\left(\omega \right)$, *x*_0_, *y*_0_ and *ϕ*. The parameters *ω*_CBP_ and *γ*_CBP_ are fitting parameters limited to the ranges (1450, 1452.5) cm^−1^ and (6,7) cm^−1^, respectively. From the fit parameters we obtain the eigenfrequencies *ω*_±_ of the coupled system using the following relation

10 $${\omega }_{\pm }=\frac{1}{2}\left({\omega }_{{{{{{\rm{PhP}}}}}}}+{\omega }_{{{{{{\rm{CBP}}}}}}}\right)\pm \frac{1}{2}{{{{{\rm{Re}}}}}}\left[\sqrt{4{\left|g\right|}^{2}+{\left[\Delta \omega -\frac{i}{2}\Delta \gamma \right]}^{2}}\right],$$

with Δ*ω* = *ω*_PhP_-*ω*_CBP_ and Δ*γ* = *γ*_PhP_-*γ*_CBP_. Equation (10) can be obtained by solving for the eigenvalues of Eqs. (4) and (5), together with the secular approximations $\left({\omega }_{{{{{{\rm{PhP}}}}}}}^{2}-{\omega }_{\pm }^{2}\right)=({\omega }_{{{{{{\rm{PhP}}}}}}}+{\omega }_{\pm })({\omega }_{{{{{{\rm{PhP}}}}}}}-{\omega }_{\pm })\approx 2{\omega }_{\pm }({\omega }_{{{{{{\rm{PhP}}}}}}}-{\omega }_{\pm })$ and $\left({\omega }_{{{{{{\rm{CBP}}}}}}}^{2}-{\omega }_{\pm }^{2}\right)=({\omega }_{{{{{{\rm{CBP}}}}}}}+{\omega }_{\pm })({\omega }_{{{{{{\rm{CBP}}}}}}}-{\omega }_{\pm })\approx 2{\omega }_{\pm }({\omega }_{{{{{{\rm{CBP}}}}}}}-{\omega }_{\pm })$.

## Supplementary information

Supplementary Information

## Data Availability

Data that support the results of this work are available upon reasonable request from the corresponding author.
