# Supplementary material for: Remote near-field spectroscopy of vibrational strong coupling between organic molecules and phononic nanoresonators
Source: Nat Commun. 2022 Nov 11;13:6850. doi: 10.1038/s41467-022-34393-4 (PMC9652397; doi:10.1038/s41467-022-34393-4)
Supplement: Supplementary file 1 — Supplementary Information [file 41467_2022_34393_MOESM1_ESM.pdf]

Supplementary Information for:

Remote near-field spectroscopy of vibrational strong coupling between organic molecules and phononic nanoresonators

Irene Dolado[1*], Carlos Maciel-Escudero[1,2*], Elizaveta Nikulina[1], Evgenii Modin[1], Francesco Calavalle[1], Shu Chen[1], Andrei Bylinkin[1], Francisco Javier Alfaro-Mozaz[3], Jiahan Li[4], James H. Edgar[4], Felix Casanova[1,5], Saül Vélez[6], Luis E. Hueso[1,5], Ruben Esteban[2,3], Javier Aizpurua[2,3], Rainer Hillenbrand[5,7#]

[1] CIC nanoGUNE BRTA, 20018 Donostia-San Sebastián, Spain
[2] Materials Physics Center, CSIC-UPV/EHU, 20018 Donostia-San Sebastián, Spain
[3] Donostia International Physics Center (DIPC), Donostia-San Sebastián, Spain
[4] Tim Taylor Department of Chemical Engineering, Kansas State University Manhattan, KS 66506 USA
[5] IKERBASQUE, Basque Foundation for Science, 48009 Bilbao, Spain
[6] IFIMAC-Condensed Matter Physics Center, Instituto Nicolás Cabrera, and Departamento de Física de la Materia Condensada, Universidad Autónoma de Madrid, Madrid, E-28049, Spain
[7] CIC nanoGUNE BRTA and Department of Electricity and Electronics, UPV/EHU, 20018 Donostia-San Sebastián, Spain

[*] Equally contributing authors
[#] Corresponding author: r.hillenbrand@nanogune.eu

Table of Contents

Supplementary Note 1. Permittivities of h-BN and CBP 3
Supplementary Note 2. Nonradiative (dark) 2nd-order Fabry-Perot mode........ 4
Supplementary Note 3. Fitting of experimental and simulated near-field spectra 7
Supplementary Note 4. Higher-order modes in the h-BN resonator 9
Supplementary Note 5. Discussion of the loop in the near-field spectra........ 11
Supplementary Note 6. Numerical simulations where the tip is modeled as a platinum conical structure........ 13
A. Simulation details........ 13
B. Tip on top of the h-BN nanoresonator........ 15
C. Tip on top of the h-BN nanoresonator half-covered with CBP 18
Supplementary Note 7. Fitting parameters of the simulated spectra 24
References for Supplementary Information 25

Supplementary Note 1. Permittivities of h-BN and CBP

In Supplementary Fig. 1 we show the in- and out-of-plane permittivities of monoisotopic (^{10}B) h-BN, $\varepsilon_\perp$ and $\varepsilon_\parallel$, respectively, as well as the permittivity of CBP.

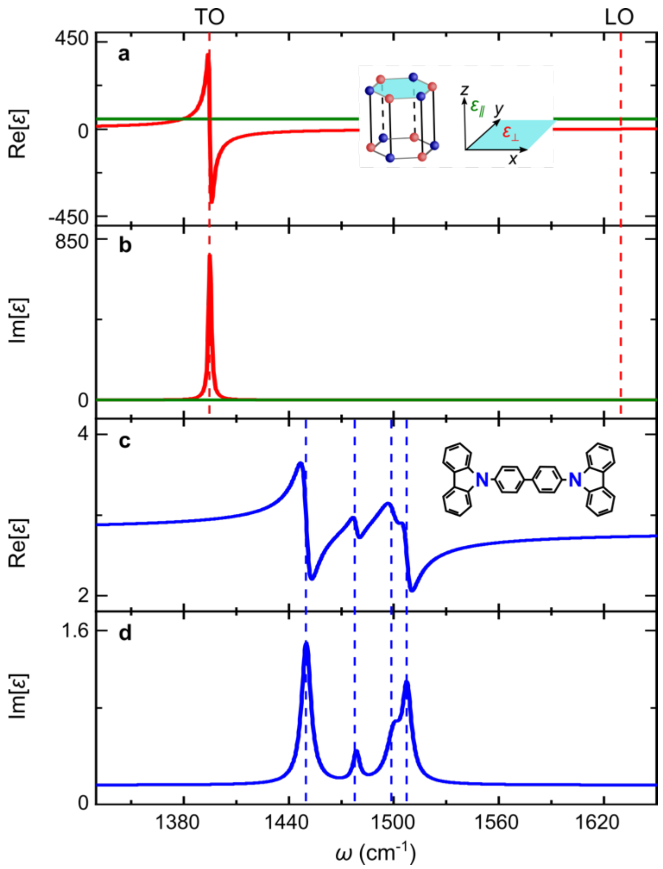

Supplementary Figure 1. **a** Real and **b** imaginary parts of the in- and out-of-plane permittivities of monoisotopic (^{10}B) h-BN, $\varepsilon_\perp$ (red curve) and $\varepsilon_\parallel$ (green curve, multiplied by 20), respectively. The red-dashed vertical lines indicate the TO and LO phonon frequencies $\omega_{\mathrm{TO},\perp} = 1395$ cm^{-1} and $\omega_{\mathrm{LO},\perp} = 1630$ cm^{-1} (Supplementary Ref. 1). **c** Real and **d** imaginary parts of the permittivity of CBP. The vertical-blue-dashed lines indicate the resonance frequencies $\omega_{0,1} = 1450$ cm^{-1}, $\omega_{0,2} = 1478.6$ cm^{-1}, $\omega_{0,3} = 1500.1$ cm^{-1} and $\omega_{0,4} = 1507.4$ cm^{-1} (Supplementary Ref. 2). Insets in panels a and c illustrate the crystal lattice structure of h-BN and the molecular structure of CBP (adapted from Supplementary Ref. 3 and https://www.ossila.com/en-eu/products/cbp), respectively.

Supplementary Note 2. Nonradiative (dark) 2nd-order Fabry-Perot mode

In this section we verify that the 2nd-order Fabry-Perot (FP) mode excited in the h-BN nanoresonator is a dark mode. To that end, we calculate the absorption cross-section $\sigma_{\rm abs}$ and the induced dipole moment $\mathbf{p}_{\rm rod}$ along the nanorod (840×250×87 nm^3) when a 1 μm Pt tip is located at the left extremity of the resonator (see schematics in Supplementary Fig. 2a). The tip and the resonator are illuminated with a p-polarized plane wave. We refer the reader to Supplementary Note 6.A, where we explain in detail the procedure to simulate the Pt tip on top of the h-BN nanoresonator.

The incident illumination, together with the tip being on top of the resonator, induce charge and current densities along the h-BN nanorod, $\rho_{\rm rod}$ and $\mathbf{J}_{\rm rod}$, respectively. Thus, to calculate the induced dipole moment in the nanorod, $\mathbf{p}_{\rm rod}$, we compute the first moment of the charge density $\rho_{\rm rod}$ according to the following equation:

$$\mathbf{p}_{\rm rod} = \iiint_V \mathbf{r}\,\rho_{\rm rod}\,\mathrm{d}^3r\,, \tag{1}$$

where d^3r is the volume element and $\mathbf{r} = (x, y, z)$ the position vector. The integration in Supplementary Eq. (1) is performed along the complete volume V of the h-BN nanorod. In addition, the induced charge $\rho_{\rm rod}$ and current $\mathbf{J}_{\rm rod}$ densities are related via the continuity equation as follows $\nabla \cdot \mathbf{J}_{\rm rod} = i\omega\rho_{\rm rod}$. Thus, by substituting the continuity equation into Supplementary Eq. (1) and integrating by parts, one finds that $\mathbf{p}_{\rm rod}$ can be written in terms of $\mathbf{J}_{\rm rod}$ as

$$\mathbf{p}_{\rm rod} = \frac{i}{\omega}\iiint_V \mathbf{J}_{\rm rod}\,\mathrm{d}^3r\,. \tag{2}$$

In practice, to calculate the induced dipole moment $\mathbf{p}_{\rm rod}$ (Supplementary Eq. (2)), we compute the total electric field $\mathbf{E}$ inside the h-BN nanorod and calculate the current density as $\mathbf{J}_{\rm rod} = -i\omega\left((\varepsilon_\perp - 1)E_x, (\varepsilon_\perp - 1)E_y, (\varepsilon_\parallel - 1)E_z\right)$, where $\varepsilon_\perp$ and $\varepsilon_\parallel$ are the in-plane and out-of-plane components of the h-BN permittivity tensor. The absorption cross-section was calculated as $\sigma_{\rm abs} = \frac{1}{S_0}\iiint_V \mathrm{Re}[\mathbf{J}_{\rm rod}^* \cdot \mathbf{E}]$, where $S_0 = \frac{|\mathbf{E}_0|^2}{2\,Z_0}$, $|\mathbf{E}_0|$ = 1 V/m is the amplitude of the incident plane wave and Z_0 is the impedance of vacuum.

In Supplementary Fig. 2b we first show the amplitude spectrum of the induced dipole moment $|\mathbf{p}_{\rm rod}|$ (red line) in the h-BN nanorod. From the $|\mathbf{p}_{\rm rod}|$ spectrum, we clearly see a peak at $\omega_{\rm l1}$ = 1419 cm^{-1}, which can be associated with the longitudinal 1st-order FP mode excited by the Pt tip in the h-BN nanoresonator. On the other hand, the absorption cross-section spectrum, $\sigma_{\rm abs}$ (blue line), exhibits strong peaks at the frequencies $\omega_{\rm l1}$ = 1419 cm^{-1}, $\omega_{\rm t2}$ = 1454 cm^{-1} and $\omega_{\rm l2}$ = 1463 cm^{-1}. The subscripts l and t stand for longitudinal and transverse modes, respectively, whereas the numbers indicate the number of nodes of the mode. To confirm the nature of these peaks, we show

the near-field plots of the z-component of the total electric field $|E_z(\omega)|$ at ω_{t2} (panel c) and ω_{l2} (panel d). In panel c we observe a mode that is localized at the edge of the nanorod with two nodes along the z-direction (transverse edge mode), whereas the near-field plot in panel d clearly reveals the field pattern of the (longitudinal) 2nd-order FP mode. From this analysis we can conclude that the Pt tip on top of the h-BN nanorod excites the 2nd-order FP mode in the resonator. More important, the 2nd-order FP mode exhibits a vanishing induced dipole moment (nonradiative) and an absorption different from zero, and thus is a dark electromagnetic mode.

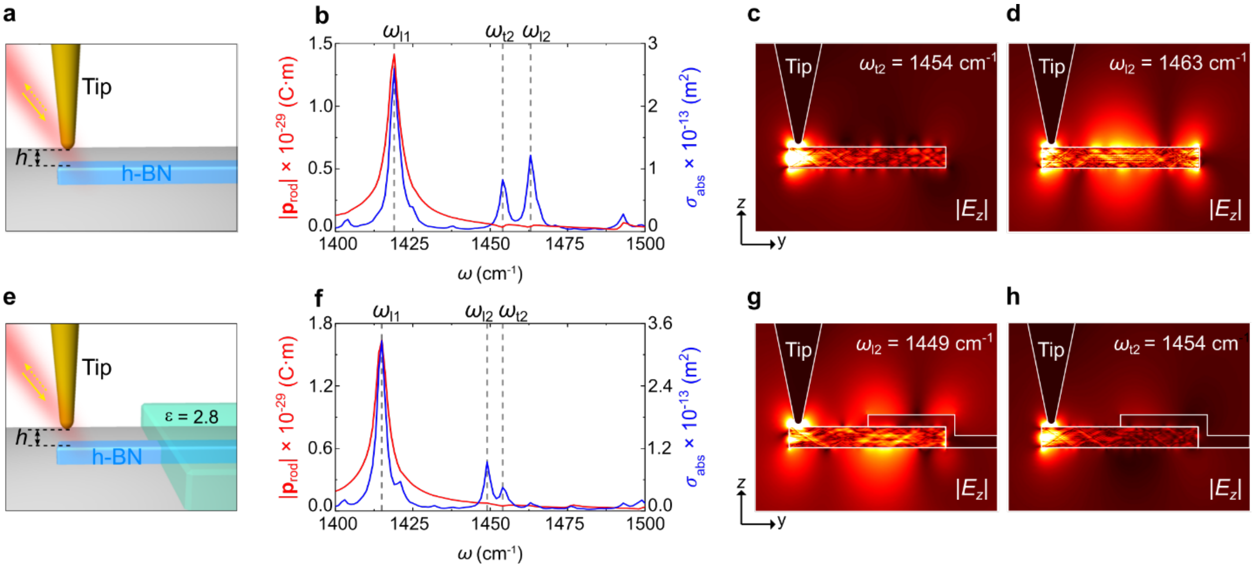

Supplementary Figure 2. **a, e** Schematics showing a Pt tip at height h above a **a** bare h-BN nanorod and **e** h-BN nanorod half-covered with a layer of permittivity $\varepsilon = 2.8$. The red lines in **b** and **f** show the amplitude of the induced dipole moment $|\mathbf{p}_{\mathrm{rod}}(\omega)|$ in the h-BN nanorod. The blue lines in **b** and **f** show the absorption cross-section of the h-BN nanorod, $\sigma_{\mathrm{rod}}(\omega)$. The field plots in panels **c**, **d**, **g** and **h** show the amplitude of the z-component of the total electric field $|E_z(\omega)|$ in the plane $x = 0$ for the frequencies $\omega_{\mathrm{t2}} = 1454$ cm^{-1} (panel c), $\omega_{\mathrm{l2}} = 1463$ cm^{-1} (panel d), $\omega_{\mathrm{l2}} = 1449$ cm^{-1} (panel g) and $\omega_{\mathrm{t2}} = 1454$ cm^{-1} (panel h). Grey-dashed lines in panels b and f mark the peak positions of the transverse edge mode, as well as the 1st- and 2nd-order FP modes, ω_{t2}, ω_{l1} and ω_{l2}, respectively. For the calculations, we use a h-BN nanorod of 840 nm length, 250 nm width and 87 nm height. 10 nm above the left nanorod extremity a Pt tip of 1000 nm height and 25 nm apex radius is placed. Tip and nanorod are illuminated with a plane wave at an angle of 60° relative to the tip axis.

To ensure that the 2nd-order FP mode remains dark after covering the h-BN nanoresonator with the CBP layer, we calculate the induced dipole moment $\mathbf{p}_{\mathrm{rod}}$ and the absorption cross-section σ_{abs} of a nanorod (840×250×87 nm^3, same dimensions as the ones used for the calculations shown in Supplementary Fig. 2b) that is half covered with a layer of permittivity $\varepsilon = 2.8$, as depicted in Supplementary Fig. 2e. We show in Supplementary Fig. 2f the amplitude of the induced dipole moment $|\mathbf{p}_{\mathrm{rod}}|$ (red line) and the absorption cross-section σ_{abs} (blue line) of the nanorod. Similarly to the bare nanorod calculation shown in Supplementary Fig. 2b, the two peaks around 1450 cm^{-1} in σ_{abs} can be attributed to the 2nd-order FP mode (peak at $\omega_{\mathrm{l2}} = 1449$ cm^{-1}) and the edge mode

(peak at $\omega_{t2} = 1454$ cm^{-1}) that is excited by the Pt tip on top of the resonator. We confirm this by plotting the near-field pattern at each frequency (see Supplementary Figs. 2g and 2h). We further find that the 2nd-order FP mode exhibits a spectral peak shift to lower frequencies as compared to the peak in the spectra obtained for the bare nanorod (compare blue curves in Supplementary Fig. 2b and Fig. 2f). We attribute this spectral peak shift to the electromagnetic loading of the h-BN nanoresonator by the layer of permittivity $\varepsilon = 2.8$. Finally, we find that the 2nd-order FP mode exhibits a vanishing induced dipole moment even for the half cover resonator, corroborating that it is a dark electromagnetic mode.

Supplementary Note 3. Fitting of experimental and simulated near-field spectra

In Supplementary Fig. 3 we show the fittings to both experimental and simulated near-field spectra from which we obtained the eigenfrequencies and coupling strengths g that are shown in Figs. 1e and 4a-b of the main text. The fitting of the spectra was performed using Eqs. (8) and (9) provided in the Methods Section of the main text. For the fittings of the simulated and experimental spectra, ω_{CBP} was fixed between 1448.5 and 1452.5 cm^{-1} and γ_{CBP} between 6 and 7 cm^{-1}. For the fittings of the simulated spectra (Supplementary Fig. 3a), we fix the minimum limit of γ_{PhP} to 3 cm^{-1} (based on the fits of Fig. 3a of the main text), and for fittings the experimental spectra we fix γ_{PhP} to a minimum value of 7 cm^{-1} (based on the fits of a half-covered h-BN nanorod whose resonance is completely detuned with respect to ω_{CBP}).

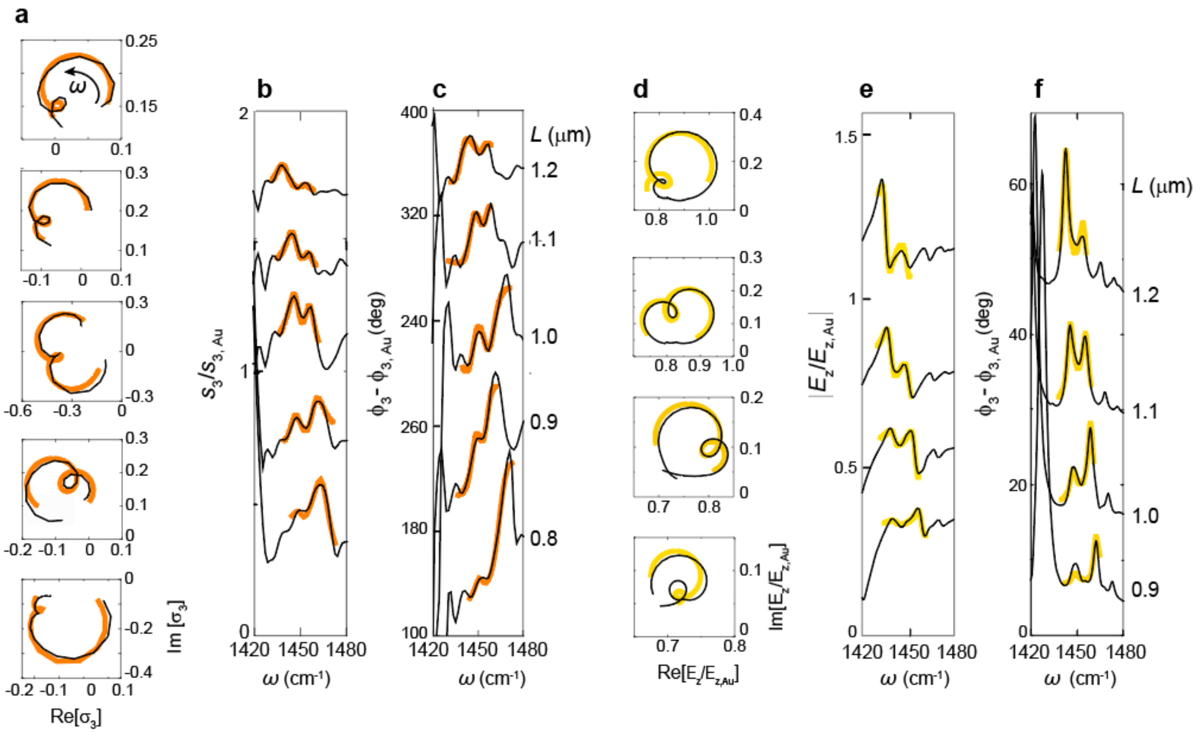

Supplementary Figure 3. **a-c** Experimental near-field spectra (black lines) of half CBP-covered h-BN nanoresonators of length L. The orange lines show fittings with the coupled harmonic oscillator model. The experimental amplitude spectra **b** are the same as the ones shown in Fig. 1d and Fig. 4a of the main text. The amplitude spectra for L = 1.0 µm is scaled by a factor of 0.7 for better visualization of data. The representation of the near-field spectra and fittings in the complex plane **a** are the same as the ones shown in Fig. 4b of the main text. The phase spectra **c** were recorded simultaneously with the amplitude spectra. **d-f** Simulated near-field spectra (black lines) of half CBP-covered h-BN nanoresonators of length L. The yellow lines show fittings with the coupled harmonic oscillator model. The simulated amplitude spectra **e** are the same as the ones shown in Fig. 1e of the main text. The simulated phase spectra are shown in **f**. The complex representation of the simulated near-field spectra is shown in **d**. All amplitude and phase spectra spectra in b, c, e and f are offset.

Supplementary Tables 1 and 2 show all fitting parameters, obtained for the simulated and experimental near-field spectra.

L (μm)	ω_{PhP}	γ_{PhP}	F_{PhP}	ω_{CBP}	γ_{CBP}	x_0	y_0	g
**0.9**	1445.6	3.0	2268	1450.5	7.0	0.85	0.04	5.5
**0.8**	1450.0	3.0	1908	1450.7	7.0	0.82	0.04	5.3
**0.7**	1455.2	3.0	1171	1450.9	6.4	0.76	0.05	5.2
**0.6**	1459.9	3.1	895	1451.0	6.0	0.71	0.05	5.5

Supplementary Table 1. Parameters obtained by fitting the simulated complex-valued spectra, shown in Supplementary Fig. 3d, to Eq. (8) of the main text. All parameters are expressed in cm^{-1}, except for x_0, y_0 (adimensional), and F_{PhP} (which is expressed in cm^{-2}).

L (μm)	ω_{PhP}	γ_{PhP}	F_{PhP}	ω_{CBP}	γ_{CBP}	x_0	y_0	g	ϕ
**1.2**	1441.2	9.4 ± 2.1	1607	1450.8	6.0 ± 1.7	0.03	0.12	5.4 ± 0.5	0.05
**1.1**	1447.8	8.9 ± 1.9	2387	1452.3	6.0 ± 1.2	-0.02	0.13	4.3 ± 0.4	0.57
**1.0**	1451.0	8.8 ± 3.0	7181	1451.4	7.0 ± 1.8	-0.14	0.05	4.6 ± 0.5	1.85
**0.9**	1454.4	10.2 ± 1.9	3731	1451.8	6.6 ± 0.3	-0.08	0.07	6.0 ± 0.1	0.00
**0.8**	1462.4	10.7 ± 1.6	5449	1452.0	7.0 ± 3.3	-0.10	-0.02	4.7 ± 0.6	3.29

Supplementary Table 2. Parameters obtained by fitting the experimental complex-valued spectra, shown in Supplementary Fig. 3a, to Eq. (9) of the main text. All parameters are expressed in cm^{-1}, except for x_0, y_0 (adimensional), ϕ (which is expressed in radians) and F_{PhP} (which is expressed in cm^{-2}). The uncertainties are the standard deviations obtained by fitting the experimental spectra.

Supplementary Note 4. Higher-order modes in the h-BN resonator

As pointed out in the main text, there are several peaks in the experimental near-field spectra (Fig. 1d), which are not seen in the numerical simulations of the amplitude spectra shown in Fig. 1e. They can be attributed to higher-order PhP modes, as we demonstrate in the following.

In Supplementary Figs. 4a-c we show the near-field spectrum of a half molecule-covered nanoresonator, whose length was chosen such that the 2nd-order FP resonance is at 1450 cm^{-1}. The tip is modelled as a point-dipole source and the z-component of the total electric field, E_z, is interpreted as the near-field signal, analogue to Fig. 1e of the main text (see also Methods Section in the main text). In the frequency range from 1430 to 1441 cm^{-1} (grey shaded region in Supplementary Fig. 4b) the amplitude spectrum $|E_z(\omega)|$ does not show clearly any resonance peak. By plotting E_z in the complex plane (Supplementary Fig. 4c), however, we see a small kink in the complex trajectory, indicating a weakly excited mode.

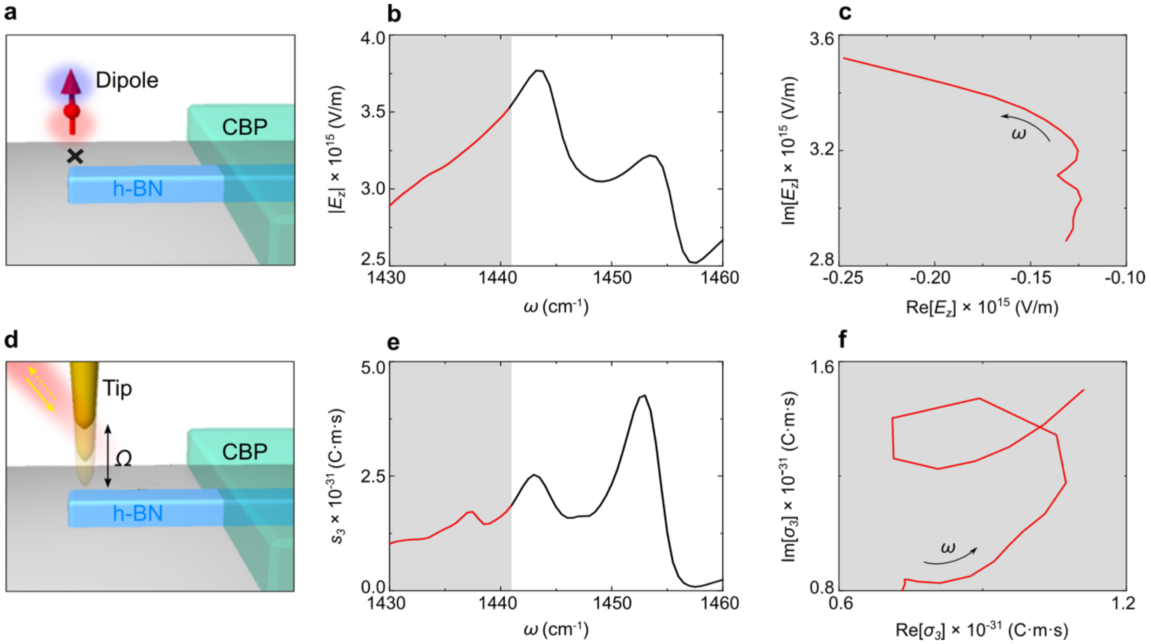

Supplementary Figure 4. a Schematics showing a point-dipole source above an h-BN nanorod of 840 nm length, 87 nm height and 250 nm width, which is half covered with a 50 nm thick CBP layer. **b** Near-field amplitude below the point dipole (at the position marked by a cross in panel a) as a function of frequency ω. The grey shaded region marks the frequency region from 1430 to 1441 cm^{-1}. **c** Near-field below the dipole source, plotted in the complex plane from 1430 to 1441 cm^{-1}. The arrow indicates increasing frequency. **d** Schematics showing a conical metal tip oscillating above the h-BN nanoresonator at frequency Ω. **e** Amplitude of the 3rd-order demodulated scattered field s_3. The grey shaded region marks the frequency region from 1430 to 1441 cm^{-1}. **f** 3rd-order demodulated scattered field σ_3, plotted in the complex plane from 1430 to 1441 cm^{-1}. The arrow indicates increasing frequency. The simulations were performed without considering the SiO_2/Si substrate.

We repeated the simulation but modelled the tip as a platinum cone (see schematics in Supplementary Fig. 4d) and calculated the complex-valued 3rd-order demodulated scattered field (σ_3, Supplementary Figs. 4e-f). For simulation details see Supplementary Note 6.A. In contrast to the near-field spectrum obtained with the point-dipole source simulation (Supplementary Fig. 4b), we observe a clear peak at around 1437 cm^{-1} in the amplitude spectrum s_3 (Supplementary Fig. 4e). We attribute this peak to a higher-order PhP mode excited in the nanoresonator. It corresponds to a loop when plotting σ_3 in the complex plane (Supplementary Fig. 4f) and to the kink observed in Supplementary Fig. 4c. We conclude that the excitation of this higher-order PhP mode is stronger for the tip being a metal cone rather than a point dipole. For that reason, the peak is seen in the experimental near-field amplitude spectrum (Fig. 1d) but not in the simulated near-field amplitude spectrum (Fig. 1e).

Supplementary Note 5. Discussion of the loop in the near-field spectra

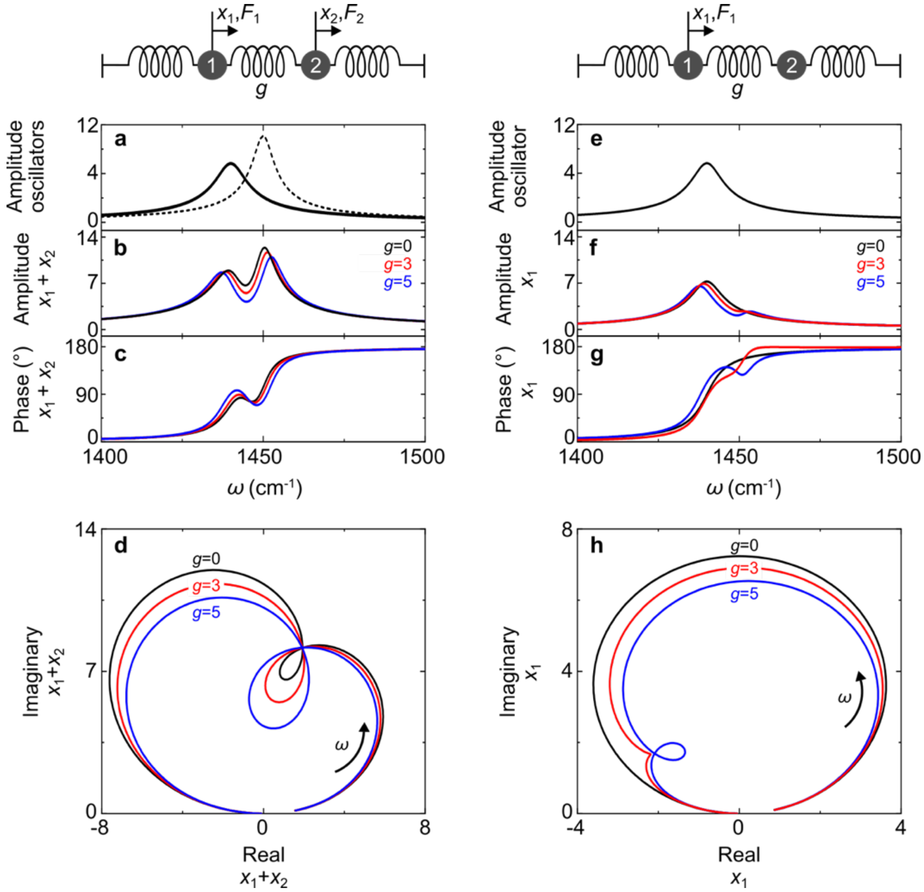

Supplementary Figure 5. Comparison of coupled mechanical oscillators. **a-d** Two oscillators labeled 1 and 2 are driven and thus displaced by distances x_1 and x_2, respectively. The complex-valued oscillation amplitude of the combined displacement $x_1 + x_2$ is analysed as a function of frequency. **a** Spectra of the oscillation amplitude of two slightly frequency-detuned, uncoupled oscillators. The first oscillator (solid curve) has eigenfrequency $\omega_1 = 1440$ cm^{-1}, damping $\gamma_1 = 9.6$ cm^{-1} and driving force $F_1 = 1 \times 10^5$ cm^{-2}. The second oscillator (dashed curve) has eigenfrequency $\omega_2 = 1450$ cm^{-1}, damping $\gamma_2 = 6.5$ cm^{-1} and driving force $F_2 = 1 \times 10^5$ cm^{-2}. **b** Amplitude and **c** phase spectra for three coupling strengths: $g = 0$ cm^{-1} (black line), 3 cm^{-1} (red line) and 5 cm^{-1} (blue line). **d** Representation of the spectra of panel b and c in the complex plane. **e-h** Only oscillator 1 is driven and only its individual complex-valued oscillation amplitude x_1 is analysed. **e** Spectrum of the oscillation amplitude of the driven oscillator (solid curve), which has eigenfrequency $\omega_1 = 1440$ cm^{-1}, damping $\gamma_1 = 9.6$ cm^{-1} and driving force $F_1 = 1 \times 10^5$ cm^{-2}. The second oscillator has eigenfrequency $\omega_2 = 1450$ cm^{-1}, damping $\gamma_2 = 6.5$ cm^{-1} and driving force $F_2 = 0$ cm^{-2}. **f** Amplitude and **g** phase spectra for the three coupling strengths: $g = 0$ cm^{-1} (black line), 3 cm^{-1} (red line) and 5 cm^{-1} (blue line). **h** Representation of the spectra of panel f and g in the complex plane.

In Supplementary Figs. 5a-d we show a situation where two oscillators are driven by the same force $F_1 = F_2$ (see inset above Supplementary Fig. 5a), which, for example, corresponds to two slightly frequency-detuned molecule resonances that are studied by far-field spectroscopy. By

probing the two-oscillators, we see a loop when the response (for instance $x_1 + x_2$) is plotted in the complex plane for all coupling strengths g, even for $g = 0$ (Supplementary Fig. 5d). Thus, the appearance of the loop does not indicate necessarily coupling between the two oscillators but can be just the consequence of a mere superposition of two resonances.

In Supplementary Figs. 5e-h we show the situation where only one oscillator is driven by the force F_1 (see inset above Supplementary Fig. 5e), which, for example, corresponds to our experiment. By probing only the driven oscillator and plotting its response, x_1, in the complex plane (Supplementary Fig. 5h), we see that the loop appears only for $g > 0$, demonstrating that the appearance of the loop reveals the coupling between the two oscillators. By reducing *g,* the loop experiences a topological transition to a kink that already indicates coupling between the two oscillators. In case of $g = 0$ we do not observe any response of the second (non-driven) oscillator, that is, neither a kink nor a loop is seen in the black trajectory.

Supplementary Note 6. Numerical simulations where the tip is modeled as a platinum conical structure

A. Simulation details

To theoretically study the influence of the metallic tip and the signal demodulation on the determination of the hybrid polariton modes, we performed numerical simulation where the tip is modelled as a conical platinum structure of 1 μm length with a 16° half angle and semispherical apex of 25 nm radius. The symmetry axis of the tip is parallel to the *z*-direction (perpendicular to the nanoresonator surface, see Fig. 1a in the main text). The h-BN nanoresonator and the CBP molecular layer were modeled in the same manner as explained in the main text (see Methods Section). In addition, the simulations with the metallic tip were performed using the scattering problem approach (implemented in the Radio Frequency Module of COMSOL Multiphysics), which allowed us to distinguish between the incident illumination (background field, $\mathbf{E}_{\text{inc}}$), and the scattered field (relative field, $\mathbf{E}_{\text{scat}}$). The incident illumination $\mathbf{E}_{\text{inc}}$ was defined as a p-polarized plane wave, incident at the tip apex, 60° relative to the symmetry axis of the tip.

The illuminated tip interacts with the nanorod via the near fields around the tip apex. This interaction results predominantly in a net vertical dipole moment induced along the conical structure, p_z. Thus, the tip-scattered field E_{scat} can be interpreted as the radiation emitted by this net dipole, which is proportional to p_z. The latter was calculated as the first moment of the surface charge density induced along the tip, ρ_s. The tip-scattered field E_{scat} can thus be related to ρ_s according to the following equation

$$E_{\text{scat}} \propto p_z = \iint_S z\, \rho_s\, \mathrm{d}^2 r, \tag{3}$$

where $\mathrm{d}^2 r$ is the surface element with outward normal unit vector $\hat{\mathbf{n}}$, z is the vertical position, $\rho_s = \varepsilon_0 \left[\mathbf{E}^{\text{out}}(S) - \mathbf{E}^{\text{in}}(S)\right] \cdot \hat{\mathbf{n}}$ and $\mathbf{E}^{\text{out}}(\mathbf{E}^{\text{in}})$ is the total electric field outside (inside) the tip. The integration in Supplementary Eq. (3) is performed along the complete surface *S* of the tip. To determine E_{scat}, we consider only the *z*-component of the induced dipole moment because p_x and p_y components are relatively small as the tip is strongly elongated in the *z*-direction.

For simplicity and to reduce the numerical calculation time, the simulations with the Pt tip were performed without considering the SiO_2/Si substrate. In all the simulations the tip-surface apex was located at coordinates ($x = 0$, $y = -L/2 + 50$ nm, $z = h$) where *h* is the height between the tip apex and the h-BN nanorod top surface. We set the origin ($x = 0$, $y = 0$, $z = 0$) at the middle of the top surface of the h-BN nanorod. In addition, we calculated the surface charge density σ_{tip} using only the electric field outside the tip, that is $\rho_s = \varepsilon_0 \mathbf{E}^{\text{out}}(S) \cdot \hat{\mathbf{n}}$. This approximation is justified by the high reflectivity of platinum at the frequency range between 1400-1500 cm^{-1}. As a consequence, the electric field inside the platinum tip is close to zero as we have verified. The permittivity of Pt was taken from Supplementary Ref. 4.

To ensure numerical convergence of the calculated induced dipole moment p_z, the mesh of the Pt tip is chosen extremely fine with a maximum element size of 60 nm. In addition, the complete structure (Pt tip, h-BN nanorod and CBP layer) is located in a homogeneous rectangular box (filled with air) of 4.2×3.75×4.35 μm^3 size. We use perfectly matched layers (PML) for the boundaries of the simulation box and free triangular elements for the nanorod mesh and free tetrahedral elements for all other structures.

To suppress background scattering signals in the experiment, the tip is oscillating normal to the surface, i.e. the distance between tip and sample is modulated. Consequently, the tip-scattered field is modulated at the oscillation frequency, $f = 2\pi\Omega$, and its harmonics. To consider tip modulation and signal demodulation in our simulations, we apply the following procedure[5–7]:

(i) We calculate the induced dipole moment (p_z, Supplementary Eq. (3)) for different tip heights h = 1, 2, 3, 4, 5, 6, 7, 8, 9, 10, 15, 20, 25, 30, 35, 40, 45 and 50 nm. We define the tip height as the minimum distance between the tip apex and the nanorod surface. For each simulation we only vary h and leave constant the rest of the parameters.

(ii) We interpolate p_z and assume the tip height to be a harmonic function of time

$$h(t) = A(1 - \cos(\Omega t)) + h_{\text{min}}, \tag{4}$$

where $A = (h_{\text{max}} - h_{\text{min}})/2$ is the tapping amplitude with h_{min} and h_{max} being the minimum and maximum tip heights used in the simulations. To closely reproduce experimental parameters, we use a tapping frequency of 265.55 kHz.

(iii) The nth-order demodulated scattered field is proportional to $\sigma_n = s_n e^{i\varphi_n}$, which is obtained by calculating the nth Fourier coefficient of the induced dipole moment:

$$\sigma_n \propto \int_0^T p_z\big(h(t)\big)e^{in\Omega t}\,\mathrm{d}t\,, \tag{5}$$

where $T = 2\pi/\Omega$ is the period of oscillation of the tip. In practice, the Fourier transform of $p_z(h(t))$ was performed using the Fourier package of Wolfram Mathematica software. For simplicity, we refer in the following to σ_n as the nth-order demodulated scattered field.

The metallic tip on top of the h-BN nanoresonator can potentially shift the frequencies and change the spectral linewidths of the resonator eigenmodes. These two modifications, together with signal demodulation, are important properties to take into consideration when extracting eigenmode frequencies and damping constants from nano-FTIR spectra. Thus, in the following section we compare the 2nd-order FP resonance excitation with both the point-dipole source (with a constant dipole moment) and the Pt tip.

B. Tip on top of the h-BN nanoresonator

In Supplementary Fig. 6 we show the simulated near-field spectra when the metal tip is placed on the left extremity of a h-BN nanoresonator (840×250×87 nm^3), whose right half is covered by a 50 nm thick layer of permittivity $\varepsilon = 2.8$ (corresponding to the permittivity of CBP without the molecular resonances). For comparison, we also show the results obtained for the tip being mimicked by a point-dipole source (illustrated in Supplementary Fig. 6a).

We start with an extended discussion of the near-field spectra obtained for the point-dipole source. Supplementary Fig. 6b shows the near-field amplitude spectrum $|E_z(\omega)|$ at 65 nm height above the h-BN resonator (position marked by a cross in Supplementary Fig. 6a). We clearly see a strong resonance peak around 1450 cm^{-1} and another much weaker peak around 1455 cm^{-1}. We associate the strong peak to the 2nd-order FP mode[8] and the weaker one to a higher-order PhP mode[9]. The asymmetry of the strong resonance peak we attribute to Fano-like interference[10,11] between the electric field produced by the (non-resonant) point-dipole source, together with the non-polaritonic near field of the resonator, and the resonant near field of the 2nd-order FP mode. For a better understanding of the FP resonance, we plot the spectrum $E_z(\omega)$ in the complex plane (Supplementary Fig. 6c) where the 2nd-order FP resonance and the Fano interference manifests as a circular trajectory with an offset, which is the typical signature of a harmonic oscillator[12].

We next discuss the near-field spectra when a conical Pt tip is placed at different heights above the left nanoresonator extremity (illustrated in Supplementary Fig. 6d). To that end we calculate the dipole moment p_z induced in the tip as described above. For the calculation, we employed the same nanorod dimensions (840×250×87 nm^3) as the one used for the point-dipole calculations (Supplementary Figs. 6b-c). The amplitude spectra $|p_z(\omega)|$ for three different tip heights h = 1 nm (red dots), 20 nm (blue dots) and 50 nm (green dot) are shown in Supplementary Fig. 6e. From the red spectra we recognize a strong peak at 1446 cm^{-1} and weaker one at 1453 cm^{-1}, which are redshifted 4 cm^{-1} and 2 cm^{-1} as compared to the near-field spectra obtained with the point-dipole source (Supplementary Fig. 6b). We attribute these spectral peak shifts to the electromagnetic loading of the h-BN nanoresonator by the Pt tip, as we show below by fitting the spectral line shapes. As in the point-dipole calculation, the Fano spectral line shape is the result of the interference between the dipole moment induced in the Pt tip by the incident illumination and the dipole moment induced in the tip by the near fields of the h-BN nanoresonator. We further find that (i) the peak positions blueshift with increasing height h and (ii) the magnitude of p_z close to the peak (around 1446 cm^{-1}) is reduced as the tip height is increased. The decrease of $|p_z|$ can be observed in Supplementary Fig. 6f, where we plot p_z in the complex plane for the three tip heights (h = 1, 20 and 50 nm). We clearly see that the radius of curvature of the circular trajectories is decreased (compare red, blue and green curves) as h is increased meaning a reduction of $|p_z|$.

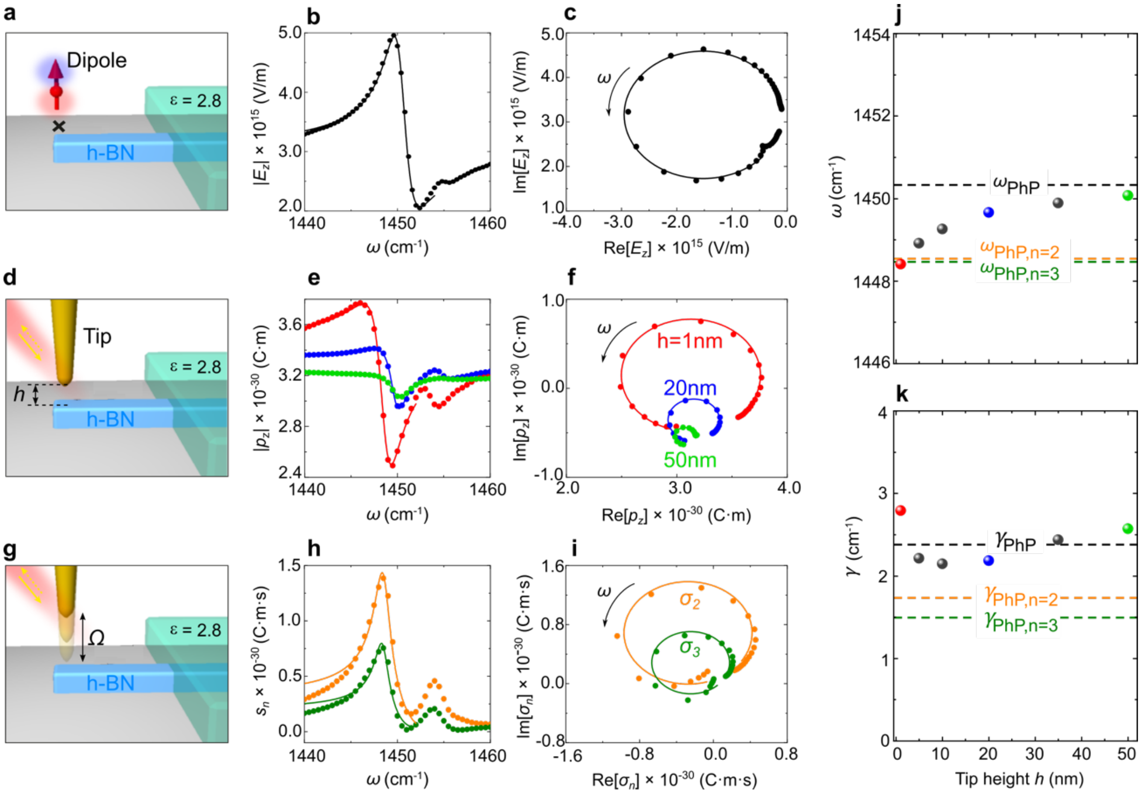

Supplementary Figure 6. **a** Schematics showing a point-dipole source above an h-BN nanorod half-covered with a layer of permittivity $\varepsilon = 2.8$. **b** Near-field amplitude $|E_z|$ below the dipole at the position marked by a cross in panel a. In the simulations, the point dipole and the evaluation point were located at coordinates ($x = 0$, $y =$ -370 nm, $z = 350$ nm) and ($x = 0$, $y =$ -370 nm, $z = 65$ nm). **c** Near-field E_z below the dipole source, plotted in the complex plane for the frequencies between 1440 and 1460 cm^{-1}. The arrow indicates increasing frequency. Dots in panels b and c are numerical results obtained from simulations. Solid lines in panels b and c represent the fitted curves using Supplementary Eq. (6). We fit the spectrum between 1440 and 1454 cm^{-1}. **d** Schematics of the Pt-tip at height h above the h-BN nanorod, which is illuminated with a broadband infrared laser beam. **e** Amplitude of the induced dipole moment $|p_z|$ in the tip for h = 1 nm (red dots), 20 nm (blue dots) and 50 nm (green dots) tip heights above the h-BN nanoresonator. **f** p_z plotted in the complex plane, from 1440 to 1452 cm^{-1}. Same color code as in panel e. Dots in panels e and f are the numerical results obtained from simulations. Solid lines in panels e and f represent fits using Supplementary Eq. (6). We fit the spectra between 1440 and 1452 cm^{-1}. **g** Schematics of the Pt tip oscillating above the h-BN nanoresonator at frequency Ω. **h** Amplitude of the 2nd- and 3rd-order demodulated scattered fields, s_2 (orange dots) and s_3 (green dots). **i** σ_2 and σ_3 plotted in the complex plane, from 1440 to 1452 cm^{-1}. Dots in panels h and i are the numerical results obtained by demodulating the complex-valued spectra $p_z(\omega)$. Solid lines in panels h and i represent fits using Supplementary Eq. (6). We fit the spectra between 1440 cm^{-1} and 1452 cm^{-1}. **j** Eigenfrequency and **k** damping of the 2nd-order FP resonance, ω_{PhP} and γ_{PhP}, for different tip heights h. Black dashed lines show the fit values obtained from panel c. Orange and green dashed lines show the fit values obtained from panel i. Dots show the fit values obtained from fitting the spectra of the induced dipole moment p_z at different tip height h = 1 nm (red dot), 20 nm (blue dot), 50 nm (green dot) and 5, 10 and 35 nm (grey dots). For all numerical calculations we use a h-BN nanorod of 840 nm length, 87 nm height and 250 nm width, which is half covered with a 50 nm thick layer of permittivity $\varepsilon = 2.8$.

To calculate the demodulated scattered field σ_n, we varied the tip height (illustrated in Supplementary Fig. 6g) in the simulations and then followed the procedure explained at the end of Supplementary Note 6A. In Supplementary Fig. 6h, we show the amplitude of the 2nd- and 3rd-order demodulated scattered fields, s_2 (orange dots) and s_3 (green dots), respectively. We see the 2nd-order PhP FP peak around 1448 cm^{-1} and the weak higher-order mode peak at 1454 cm^{-1}. As typical in s-SNOM and nano-FTIR spectroscopy, the amplitude of σ_n decrease with increasing demodulation order. We also see that the resonance peaks remain asymmetric, which we attribute to the interference between the nanoresonator modes and the near fields produced by the non-resonant near-fields of the h-BN resonator. For completeness, we plot in Supplementary Fig. 6i σ_2 (orange dots) and σ_3 (green dots) in the complex plane, where we see the circular trajectory representing the 2nd-order PhP FP mode.

To obtain the eigenfrequency and damping constant of the 2nd-order PhP FP mode, we performed a complex-valued fitting (see solid lines in Supplementary Figs. 6b, 6c, 6e, 6f, 6h and 6i) to the spectra shown in Supplementary Fig. 6. We fit the following three spectra: (i) the near-field $E_z(\omega)$ for the point-dipole simulation, (ii) the dipole moment $p_z(\omega)$ induced in the Pt tip at different tip heights and (iii) the second, σ_2, and third order, σ_3, demodulated scattered fields. We fit the spectra to the following equation

$$\hat{x}_{\mathrm{PhP}}(\omega) = \frac{\hat{F}_{\mathrm{PhP}}(\omega)}{\omega_{\mathrm{PhP}}^2 - \omega^2 - i\gamma_{\mathrm{PhP}}\omega} + x_0 + y_0 i. \tag{6}$$

The parameters $\hat{x}_{\mathrm{PhP}}(\omega)$, $\hat{F}_{\mathrm{PhP}}(\omega)$, ω_{PhP}, γ_{PhP}, x_0, y_0 are defined in the Methods Section of the main text.

In Supplementary Fig. 6j we show the values of ω_{PhP} extracted from the fittings. As a reference, we first fit (solid line in Supplementary Fig. 6c) the near-field spectrum $E_z(\omega)$ obtained from the point-dipole calculation (dots in Supplementary Fig. 6c). The fit value ω_{PhP} = 1450.3 cm^{-1} is indicated in Supplementary Fig. 6j by a black dashed line. We next fit (solid lines) the complex spectra $p_z(\omega)$ for different tip heights (induced dipole moment in the Pt tip, dots in Supplementary Fig. 6f) obtained from the simulation of the Pt tip on top of the resonator. The values of ω_{PhP} are represented in Supplementary Fig. 6j by the dots. When the tip is far from the nanoresonator, we find that ω_{PhP} is close to the fit value extracted from the $E_z(\omega)$ spectrum (compare the black dashed line and green dot). When the height h of the tip above the nanoresonator is reduced, ω_{PhP} shift to lower frequencies, which can be explained by loading of the nanoresonator by the metallic tip. Fitting the 2nd- and 3rd-order demodulated scattered field spectra, $\sigma_2(\omega)$ and $\sigma_3(\omega)$ (dots in Supplementary Fig. 6i), respectively, we find the same value of ω_{PhP} for both demodulation orders. The fit values are $\omega_{\mathrm{PhP},n=2}$ = $\omega_{\mathrm{PhP},n=3}$ = 1448.5 cm^{-1}, which we indicate by an orange and green dashed lines in Supplementary Fig. 6j. Notice that $\omega_{\mathrm{PhP},n=2}$ and $\omega_{\mathrm{PhP},n=3}$ are only 2 cm^{-1} shifted with respect to the fit value obtained from the $E_z(\omega)$ spectrum.

From the fittings we also extract the damping constant γ_{PhP} (Supplementary Fig. 6k). As a reference, we first show the fit value extracted from the near-field spectrum $E_z(\omega)$ (shown in Supplementary Fig. 6c). The fit value γ_{PhP} = 2.4 cm^{-1} is indicated in Supplementary Fig. 6k by a black dashed line. We next show the fit values extracted from the complex spectra $p_z(\omega)$ for different tip heights (induced dipole moment in the Pt tip, Supplementary Fig. 6f) obtained from the simulation of the Pt tip on top of the resonator. The fit values of γ_{PhP} are represented in Supplementary Fig. 6k by the dots. We find fit values within the range (2.1, 2.8) cm^{-1}, which differ at most 0.4 cm^{-1} with respect to the fit value obtained from the $E_z(\omega)$ spectrum. Fitting the 2nd- and 3rd-order demodulated scattered field spectra, $\sigma_2(\omega)$ and $\sigma_3(\omega)$ (shown in Supplementary Fig. 6i), respectively, we find a smaller damping constant compared to that obtained by fitting the non-demodulated spectrum of $p_z(\omega)$. The fit values are $\gamma_{\text{PhP},n=2}$ = 1.7 cm^{-1} (0.6 cm^{-1} shifted with respect to the fit value obtained from the $E_z(\omega)$ spectrum) for $\sigma_2(\omega)$ spectrum and $\gamma_{\text{PhP},n=3}$ = 1.5 cm^{-1} (0.9 cm^{-1} shifted with respect to the fit value obtained from the $E_z(\omega)$ spectrum) for $\sigma_3(\omega)$ spectrum. In Supplementary Fig. 6k, we indicate the value of $\gamma_{\text{PhP},n=2}$ by an orange dashed line and the value of $\gamma_{\text{PhP},n=3}$ by a green dashed line.

To summarize this section, we conclude that the Pt tip on top of the h-BN nanoresonator leads to negligible resonance shift and the damping constant obtained by fitting the different non-demodulated spectra (shown in Supplementary Figs. 6c and 6f) reveals small variations for the 2nd-order PhP FP resonance. On the other hand, fitting the demodulated scattered field spectra with a simple harmonic oscillator model yields damping constants which are underestimated. Thus, care must be taken when extracting damping constants from nano-FTIR spectra.

C. Tip on top of the h-BN nanoresonator half-covered with CBP

In this section, we analyze the influence of the metallic tip on top of the nanoresonator that is half covered with molecules. To that end, we show the simulated near-field spectra when the metallic tip is placed on the left extremity of a h-BN nanoresonator (840×250×87 nm^3, same dimensions as the ones used for the calculations shown in Supplementary Fig. 6), whose right half is covered by a 50 nm thick CBP layer. The length of the nanorod was chosen such that the 2nd-order PhP FP resonance is approximately tuned to the CBP molecular resonance at ω_{CBP} =1450 cm^{-1}. For comparison, we first show the results obtained for the tip being mimicked by a point-dipole source (illustrated in Supplementary Fig. 7a).

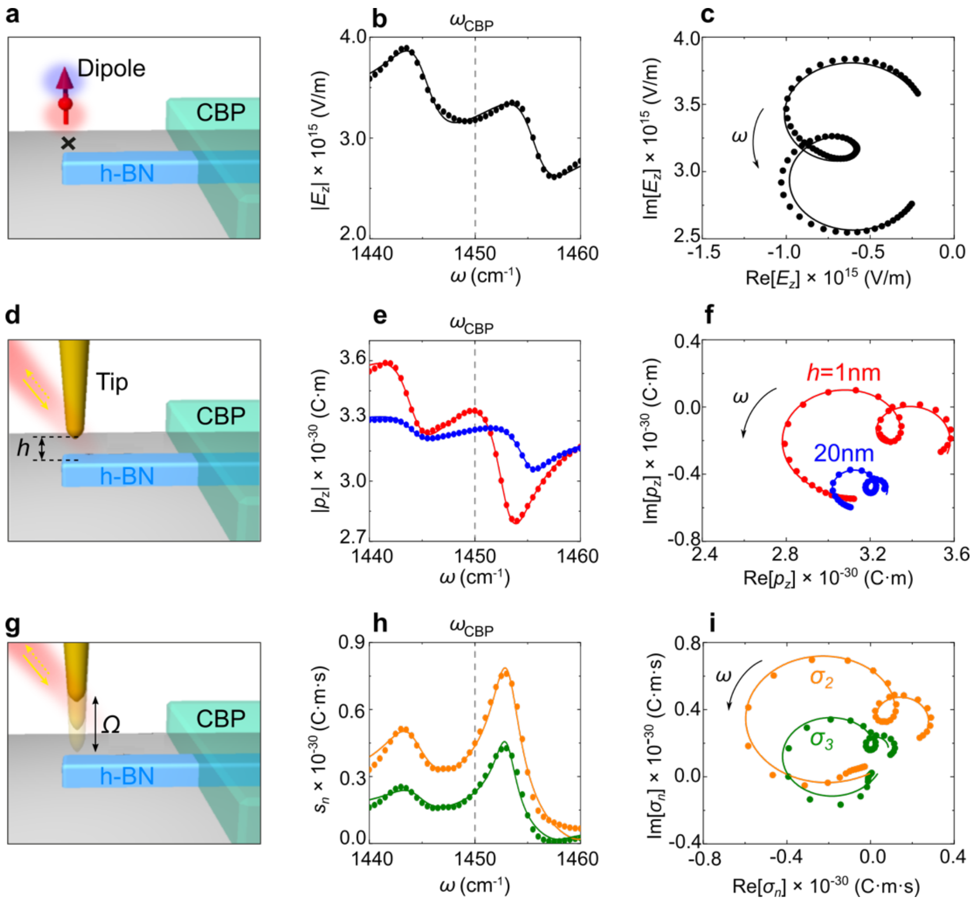

Supplementary Figure 7. A Schematics showing a point-dipole source above an h-BN nanorod half-covered by a CBP molecular layer. **b** Near-field amplitude $|E_z|$ below the dipole at the position marked by a cross in panel a. In the simulations, the point dipole and the evaluation point where located at coordinates (x = *0*, y = -370 nm, z = 350 nm) and (x = *0*, y = -370 nm, z = 65 nm). **c** Near-field E_z below the point-dipole source, plotted in the complex plane for the frequency range between 1440-1460 cm^{-1}. The arrow indicates increasing frequency. Dots in panels b and c are the numerical results obtained from simulations. Solid lines in panels b and c show the fitted curves using Supplementary Eq. (7). **d** Schematics of the Pt-tip at height h above the h-BN nanorod, which is illuminated with a broadband infrared laser beam. **e** Amplitude of the induced dipole moment p_z calculated for 1 nm (red line) and 20 nm (blue line) tip height. **f** p_z plotted in the complex plane for the frequency range between 1440 and 1460 cm^{-1}. The color code is the same as the one used in panel e. The arrow indicates increasing frequency. Dots in panels e and f are the numerical results obtained from simulations. Solid lines in panels e and f show the fitted curves using Supplementary Eq. (7). **g** Schematics of the Pt tip oscillating on top of the h-BN nanoresonator at frequency Ω. **h** Amplitude of the 2nd- and 3rd-order demodulated scattered fields, s_2 (orange dots) and s_3 (green dots). **i** σ_2 and σ_3 plotted in the complex plane for the frequencies between 1440 and 1460 cm^{-1}. The arrow indicates increasing frequency. Dots in panels h and i are the numerical results obtained by demodulating the complex spectra $p_z(\omega)$. Solid lines in panels h and i represent the fitted curves using Supplementary Eq. (7). The fittings shown in panels b, c, e, f, h and i were performed in the frequency range between 1440 cm^{-1} and 1460 cm^{-1}. For all numerical calculations we use a nanorod of 840 nm length, 87 nm height and 250 nm width.

We begin our analysis by discussing the near-field spectra obtained for the point-dipole source. Supplementary Fig. 7b shows the near-field amplitude spectrum $|E_z(\omega)|$ below the point dipole at 65 nm height above the h-BN nanoresonator (position marked by a cross in Supplementary Fig. 7a). By comparing the near-field spectra shown in Supplementary Figs. 6b and 7b, we see that the amplitude peak of the 2nd-order PhP FP resonance splits into two peaks, one around 1443.5 cm^{-1} and the other one around 1453.5 cm^{-1}. The splitting is a consequence of the coupling between the nanoresonator mode and molecular vibrations. For a better understanding of the nanoresonator-molecule coupling, we plot in Supplementary Fig. 7c the spectrum $E_z(\omega)$ in the complex plane, where we recognize the appearance of a small loop (compare Supplementary Figs. 6c and 7c). As discussed in the main text, the appearance of the loop in the complex plane is a consequence of the nanoresonator mode being coupled to the molecular vibrations.

We next discuss the near-field spectra when a conical Pt tip is placed at different heights above the left nanoresonator extremity (illustrated in Supplementary Fig. 7d). To that end, we calculated the dipole moment p_z induced in the tip as described in Supplementary Note 6A. The amplitude spectra $|p_z(\omega)|$ for two different tip heights h = 1 nm (red dots) and 20 nm (blue dots) are shown in Supplementary Fig. 7e. From the red spectra we recognize two peaks, one at 1442 cm^{-1} and one at 1450 cm^{-1}, which are shifted 1.5 cm^{-1} and 3.5 cm^{-1} as compared to the near-field spectra obtained with the point-dipole source (Supplementary Fig. 7b). We attribute these spectral peak shifts to the electromagnetic loading of the h-BN nanoresonator by the Pt tip, which we verify below by fitting the spectra. Similarly to the point-dipole calculation shown in Supplementary Fig. 7b, the appearance of two peaks and one dip around ω_{CBP} in the $|p_z(\omega)|$ spectrum is the result of the coupling between the nanoresonator mode and molecular vibrations. We further find that the position of the peaks blueshift with increasing height h. By plotting p_z in the complex plane (see Supplementary Fig. 7f) we again observe a small loop indicating the nanoresonator-molecule coupling.

To calculate the demodulated scattered field, we varied the tip height (illustrated in Supplementary Fig. 7g) in the simulations and then followed the procedure explained at the end of Supplementary Note 6A. In Supplementary Fig. 7h we show the amplitude of the 2nd- and 3rd-order demodulated scattered fields, s_2 (orange dots) and s_3 (green dots), respectively. We clearly see the two peaks, one around 1443 cm^{-1} and the other one around 1453 cm^{-1}. For completeness, we plot in Supplementary Fig. 7i the demodulated scattered fields σ_2 and σ_3 in the complex plane. In this complex representation we can see the formation of the characteristic loop indicating the nanoresonator-molecule coupling.

To obtain the eigenfrequencies and damping constants of the hybrid modes, we performed a complex-valued fitting (see solid lines in Supplementary Figs. 7b, 7c, 7e, 7f, 7h and 7i) to the spectra shown in Supplementary Fig. 7. We fit the following three spectra: (i) the near- field $E_z(\omega)$ for the point-dipole simulation, (ii) the dipole moment $p_z(\omega)$ induced in the Pt tip at different tip heights and (iii) the second, $\sigma_2(\omega)$, and third order, $\sigma_3(\omega)$, demodulated scattered fields. We fit the spectra using the following equation (analogous to Eq. (9) discussed in the main text)

$$\hat{x}_{\mathrm{sim}}(\omega) = \hat{x}_{\mathrm{PhP}}(\omega) * e^{i\theta} + x_0 + y_0 i, \tag{7}$$

which was derived from the two coupled harmonic oscillator model described in the Methods Section (Eqs. (6)-(7)). The parameters $\hat{x}_{\mathrm{PhP}}(\omega)$, $\hat{x}_{\mathrm{sim}}(\omega)$, x_0 and y_0 are defined in the Methods Section of the main text. We multiply $\hat{x}_{\mathrm{PhP}}(\omega)$ by the complex factor $e^{i\theta}$ that accounts for the additional phase gained by not normalizing the simulated spectra.

In Supplementary Figs. 8a-8b we show the values of ω_{PhP} and ω_{CBP} extracted from the fittings. As a reference, we first fit (solid line in Supplementary Fig. 7c) the near-field spectrum $E_z(\omega)$ obtained from the point-dipole calculation (dots in Supplementary Fig. 7c). The fit values are ω_{PhP} = 1450.2 cm^{-1} and ω_{CBP} = 1450.3 cm^{-1} indicated in Supplementary Figs. 8a-8b by the black dashed lines. We next fit (solid lines in Supplementary Fig. 7f) the complex-valued spectra $p_z(\omega)$ (induced dipole moment in the Pt tip; dots in Supplementary Fig. 7f) obtained from the simulation of the Pt tip on top of the resonator that is half covered with the CBP layer. The values of ω_{PhP} and ω_{CBP} are represented in Supplementary Figs. 8a-8b by the dots. When the tip is far from the nanoresonator, we find that ω_{PhP} and ω_{CBP} are close to the fit values extracted from the $E_z(\omega)$ spectrum (compare the black dashed lines and the grey dots at h = 35 nm). When the height h of the tip above the nanoresonator is reduced, ω_{PhP} and ω_{CBP} shift to lower frequencies.

Fitting the 2nd- and 3rd-order demodulated scattered field spectra, $\sigma_2(\omega)$ and $\sigma_3(\omega)$ (shown in Supplementary Fig. 7i), respectively, we find the following fit values: $\omega_{\mathrm{PhP},n=2}$ = 1449.8 cm^{-1}, $\omega_{\mathrm{PhP},n=3}$ = 1450.3 cm^{-1}, which we indicate by an orange and green dashed lines in Supplementary Fig. 8a, and $\omega_{\mathrm{CBP},n=2}$ = 1447.3 cm^{-1}, $\omega_{\mathrm{CBP},n=3}$ = 1447 cm^{-1}, which we indicate by an orange and green dashed lines in Supplementary Fig. 8b. Notice that $\omega_{\mathrm{PhP},n=2}$ and $\omega_{\mathrm{PhP},n=3}$ differ at most 0.4 cm^{-1} with respect to the fit value obtained from the $E_z(\omega)$ spectrum. On the other hand, the fit values $\omega_{\mathrm{CBP},n=2}$ and $\omega_{\mathrm{CBP},n=3}$ differ at most 3.5 cm^{-1} with respect to the fit value obtained from the $E_z(\omega)$ spectrum.

From the fittings we also extracted the damping constants γ_{PhP} and γ_{CBP}, which are plotted in the Supplementary Figs. 8c-8d. As a reference, we first show the fit value extracted from the near-field spectra $E_z(\omega)$ (shown in Supplementary Fig. 7c). The fit values γ_{PhP} = 2.3 cm^{-1} and γ_{CBP} = 7.6 cm^{-1} are indicated in Supplementary Figs. 8c-8d by a black dashed line. The sum of the damping constants extracted from the near-field spectra $E_z(\omega)$ is $\gamma_{\mathrm{PhP}} + \gamma_{\mathrm{CBP}}$ = 9.9 cm^{-1}. We next show the fit values extracted from the complex spectra $p_z(\omega)$ (induced dipole moment in the Pt tip, Supplementary Fig. 7f) obtained from the simulation of the Pt tip on top of the resonator. The fit values of γ_{PhP} and γ_{CBP} are represented in Supplementary Figs. 8c-8d by the dots. We find fit values for γ_{PhP} and γ_{CBP} within the range (2.5, 3.0) cm^{-1} and (5.3, 7.0) cm^{-1}, respectively, which differ at most 2.3 cm^{-1} from the fit values obtained from the $E_z(\omega)$ spectrum. Consequently, the sum of the damping constants lies within the range (7.8, 10.0) cm^{-1} which differ from the value $\gamma_{\mathrm{PhP}} + \gamma_{\mathrm{CBP}}$ = 9.9 cm^{-1} by 2.1 cm^{-1} (21%). Fitting the demodulated scattered fields spectra $\sigma_2(\omega)$ and $\sigma_3(\omega)$ (shown in Supplementary Fig. 7i), we find a smaller damping constant for γ_{PhP} than

that obtained by fitting the non-demodulated spectrum of $p_z(\omega)$. In Supplementary Fig. 8c we indicate the value of $\gamma_{\mathrm{PhP},n=2}$ by an orange dashed line and the value of $\gamma_{\mathrm{PhP},n=3}$ by a green dashed line ($\gamma_{\mathrm{PhP},n=2} = \gamma_{\mathrm{PhP},n=3} = 0.4$ cm^{-1}). We also plot the damping constants of the CBP oscillator in Supplementary Fig. 8d by orange and green dashed lines, respectively ($\gamma_{\mathrm{CBP},n=2}$ = 6.7 cm^{-1} and $\gamma_{\mathrm{CBP},n=3}$ = 5.9 cm^{-1}). We find that the sum of the damping constants $\gamma_{\mathrm{PhP},n=2} + \gamma_{\mathrm{PhP},n=2} = 7.1$ cm^{-1} and $\gamma_{\mathrm{PhP},n=3} + \gamma_{\mathrm{CBP},n=3} = 6.3$ cm^{-1} differ from the value $\gamma_{\mathrm{PhP}} + \gamma_{\mathrm{CBP}} = 9.9$ cm^{-1} around 2.8 cm^{-1} (28%) and 3.6 cm^{-1} (36%), respectively, corresponding to $\gamma_{\mathrm{PhP}} + \gamma_{\mathrm{CBP}} = 1.39(\gamma_{\mathrm{PhP},n=2} + \gamma_{\mathrm{CBP},n=2})$ and $\gamma_{\mathrm{PhP}} + \gamma_{\mathrm{CBP}} = 1.56(\gamma_{\mathrm{PhP},n=3} + \gamma_{\mathrm{CBP},n=3})$.

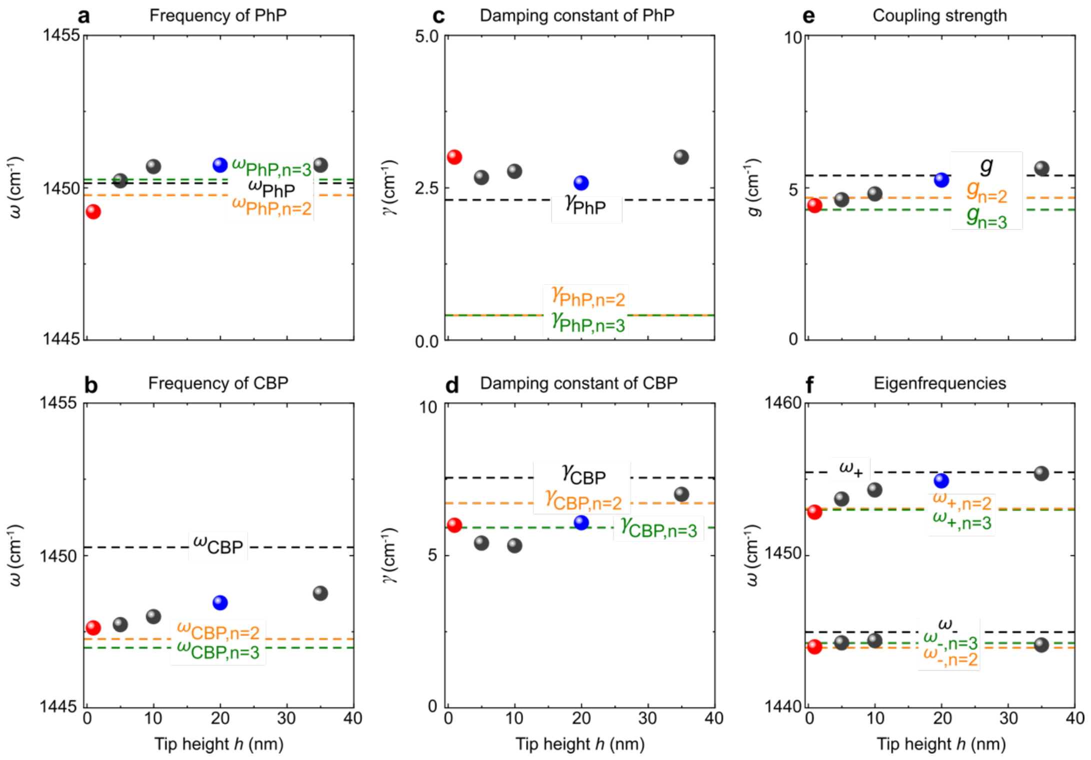

Supplementary Figure 8. Fitted values of **a** eigenfrequency of the 2nd-order FP resonance (ω_{PhP}). **b** Eigenfrequency of CBP molecular resonance (ω_{CBP}). **c** damping constant of the 2nd-order FP resonance, γ_{PhP}. **d** CBP damping constant, γ_{CBP}. **e** coupling strength g and **f** eigenfrequencies of the coupled system, $\omega_{\pm}$, as a function of the tip height h. The fittings were performed using Supplementary Eq. (7). The eigenfrequencies $\omega_{\pm}$ were obtained with Eq. (10) of the main text. Black dashed lines: fitted values obtained from the spectra plotted in Supplementary Fig. 7c. Orange and green dashed lines: fitted values obtained from the spectra plotted in Supplementary Fig. 7i. Dots: fitted values obtained from the spectra of $p_z(\omega)$ at different tip height h = 1 nm (red dot), 20 nm (blue dot) and 5, 10 and 35 nm (grey dots).

From the fittings we also extract the coupling strength g and then use Eq. (10) of the main text to calculate the eigenfrequencies $\omega_{\pm}$. The results are shown in Supplementary Figs. 8e-8f. When the tip is far from the nanoresonator, we find that g extracted from the $p_z(\omega)$ spectra is close to the fit

value $g = 5.4$ cm^{-1} extracted from the $E_z(\omega)$ spectrum (compare the black dashed line and the grey dots at h = 35 nm in Supplementary Fig. 8e). When the height h of the tip above the nanoresonator is reduced, g becomes smaller. We find fit values for g within the range (4.4, 5.7) cm^{-1}, which differ at most 1 cm^{-1} (19%) from the fit values obtained from the $E_z(\omega)$ spectrum. Fitting the 2nd- and 3rd-order demodulated scattered field spectra, $\sigma_2(\omega)$ and $\sigma_3(\omega)$ (shown in Supplementary Fig. 7i), respectively, we find coupling strength values of $g_{n=2} = 4.7$ cm^{-1} and $g_{n=3}$ = 4.3 cm^{-1}, which differ at most 1.1 cm^{-1} (21%) from the fit value obtained from the $E_z(\omega)$ spectrum, corresponding to $g = 1.26 g_{n=3}$. On the other hand, we find similar values for the eigenfrequencies ω_+ and ω_- obtained from the $E_z(\omega)$ spectrum (Supplementary Fig. 7c), from the $p_z(\omega)$ spectra (Supplementary Fig. 7f) and from $\sigma_2(\omega)$ and $\sigma_3(\omega)$ spectra (Supplementary Fig. 7i). Most importantly, we find that ω_+ and ω_- obtained from $\sigma_2(\omega)$ and $\sigma_3(\omega)$ spectra, differ at most 2.5 cm^{-1} from the eigenfrequency values obtained from the $E_z(\omega)$ spectrum.

To summarize this section, we conclude that the conical Pt tip (non-oscillating) on top of the nanoresonator-molecule coupled system leads (in comparison to the point-dipole calculation) to (i) negligible resonance shifts, (ii) changes of the sum of the spectral linewidth of at most 21% and (iii) changes in the coupling strength g of at most 19%. When we fit the demodulated scattered field spectra (tip is oscillating), we find (in comparison to the point-dipole calculation) (i) negligible resonance shifts and (ii) a change in the coupling strength*s* of at most 21%. Thus, we conclude that demodulation of the scattered field spectra does not modify substantially the eigenmode frequencies and coupling strengths. However, for the sum of the damping constants γ_{PhP} and γ_{CBP} we obtained fit values that differ around 36% from the fit value obtained from the $E_z(\omega)$ spectrum. Therefore, and similarly to the non-covered resonator case, we conclude that fitting the demodulated spectra with a two coupled harmonic oscillator model yields underestimated dampings. For that reason, care must be taken for example to obtain quality factor from near-field spectra or for the evaluation of the formal strong coupling criteria where coupling strengths are compared with the resonator, γ_{PhP}, and molecule, γ_{CBP}, dampings.

Supplementary Note 7. Fitting parameters of the simulated spectra

Supplementary Tables 3, 4 and 5 show all fitting parameters obtained for the $E_z(\omega)$, $p_z(\omega)$ and demodulated spectra shown in Supplementary Figs. 7c, 7f and 7i.

ω_{PhP}	γ_{PhP}	ω_{CBP}	γ_{CBP}	g	ω_+	ω_-	$\gamma_{\mathrm{PhP}} + \gamma_{\mathrm{CBP}}$
1450.2	2.3	1450.3	7.6	5.4	1455.5	1445.0	9.9

Supplementary Table 3. Parameters obtained by fitting the simulated complex-valued spectra, shown in Supplementary Fig. 7c (point-dipole source above an h-BN nanorod half-covered by a CBP molecular layer). All parameters are expressed in cm^{-1}.

h (nm)	ω_{PhP}	γ_{PhP}	ω_{CBP}	γ_{CBP}	g	ω_+	ω_-	$\gamma_{\mathrm{PhP}} + \gamma_{\mathrm{CBP}}$
**1**	1449.2	3.0	1447.6	6.0	4.4	1452.8	1444.0	9.0
**5**	1450.2	2.7	1447.7	5.4	4.6	1453.7	1444.3	8.1
**10**	1450.7	2.8	1448.0	5.3	4.8	1454.3	1444.4	8.1
**20**	1450.7	2.6	1448.4	6.1	5.2	1454.9	1444.3	8.6
**35**	1450.7	3.0	1448.8	7.0	5.6	1455.4	1444.1	10.0

Supplementary Table 4. Parameters obtained by fitting the simulated complex-valued spectra, shown in Supplementary Fig. 7f (static tip above an h-BN nanorod half-covered by a CBP molecular layer). All parameters are expressed in cm^{-1}.

Order harmonic	ω_{PhP}	γ_{PhP}	ω_{CBP}	γ_{CBP}	g	ω_+	ω_-	$\gamma_{\mathrm{PhP}} + \gamma_{\mathrm{CBP}}$
**O2**	1449.8	0.4	1447.3	6.7	4.7	1453.1	1443.9	7.1
**O3**	1450.3	0.4	1447.0	5.9	4.3	1453.0	1444.3	6.3

Supplementary Table 5. Parameters obtained by fitting the simulated complex-valued demodulated spectra, shown in Supplementary Fig. 7i (oscillating Pt tip above an h-BN nanorod half-covered by a CBP molecular layer). All parameters are expressed in cm^{-1}.

References for Supplementary Information

1. Dolado, I. *et al.* Nanoscale Guiding of Infrared Light with Hyperbolic Volume and Surface Polaritons in van der Waals Material Ribbons. *Advanced Materials* **32**, 1906530 (2020).
2. Bylinkin, A. *et al.* Real-space observation of vibrational strong coupling between propagating phonon polaritons and organic molecules. *Nat Photonics* **15**, 197–202 (2021).
3. Govyadinov, A. A. *et al.* Probing low-energy hyperbolic polaritons in van der Waals crystals with an electron microscope. *Nat Commun* **8**, 95 (2017).
4. Rakic´, A. D., Rakic´, R., Djuriš, A. B., Elazar, J. M. & Majewski, M. L. Optical properties of metallic films for vertical-cavity optoelectronic devices. *Appl. Opt.* **37**, 5271–5283 (1998).
5. Chen, X. *et al.* Rapid simulations of hyperspectral near-field images of three-dimensional heterogeneous surfaces. *Opt Express* **29**, 39648 (2021).
6. Maissen, C., Chen, S., Nikulina, E., Govyadinov, A. & Hillenbrand, R. Probes for Ultrasensitive THz Nanoscopy. *ACS Photonics* **6**, 1279–1288 (2019).
7. Mooshammer, F. *et al.* Quantifying Nanoscale Electromagnetic Fields in Near-Field Microscopy by Fourier Demodulation Analysis. *ACS Photonics* **7**, 344–351 (2020).
8. Alfaro-Mozaz, F. J. *et al.* Nanoimaging of resonating hyperbolic polaritons in linear boron nitride antennas. *Nat Commun* **8**, 15624 (2017).
9. Autore, M. *et al.* Enhanced Light–Matter Interaction in 10B Monoisotopic Boron Nitride Infrared Nanoresonators. *Adv Opt Mater* **9**, 2001958 (2021).
10. Nikitin, A. Y. *et al.* Real-space mapping of tailored sheet and edge plasmons in graphene nanoresonators. *Nat. Photonics* **10**, 239–243 (2016).
11. Miroshnichenko, A. E., Flach, S. & Kivshar, Y. S. Fano resonances in nanoscale structures. *Rev. Mod. Phys.* **82**, 2257–2298 (2009).
12. Jurna, M. *et al.* Visualizing Resonances in the Complex Plane with Vibrational Phase Contrast Coherent Anti-Stokes Raman Scattering. *Anal. Chem.* **82**, 7656–7659 (2010).
